# Anthropogenic change decouples a freshwater predator’s density feedback

**DOI:** 10.1038/s41598-023-34408-0

**Published:** 2023-05-10

**Authors:** J. S. Sinclair, R. Briland, M. E. Fraker, J. M. Hood, K. T. Frank, M. D. Faust, C. Knight, S. A. Ludsin

**Affiliations:** 1 Department of River Ecology and Conservation, Senckenberg Research Institute and Natural History Museum, Clamecystraße 12, 63571 Gelnhausen, Hesse, Germany; 2 Aquatic Ecology Laboratory, The Ohio State University, 1314 Kinnear Rd, Columbus, OH 43221 USA; 3 Ohio Environmental Protection Agency, 50 W. Town St. Suite 700, Columbus, OH 43215 USA; 4 Cooperative Institute for Great Lakes Research and Michigan Sea Grant, School for Environment and Sustainability, University of Michigan, 4840 S. State, Ann Arbor, MI 48108 USA; 5 Translational Data Analytics Institute, The Ohio State University, 1760 Neil Ave, Columbus, OH 43210 USA; 6 Ocean and Ecosystem Sciences Division, Bedford Institute of Oceanography, Dartmouth, NS B2Y 4A2 Canada; 7 Department of Biology, Queen’s University, Kingston, ON K7L 3N6 Canada; 8 Ohio Department of Natural Resources, Division of Wildlife, Sandusky Fisheries Research Station, 305 East Shoreline Drive, Sandusky, OH 44870 USA; 9 Ohio Department of Natural Resources, Division of Wildlife, Fairport Fisheries Research Unit, 1190 High Street, Fairport Harbor, OH, 44077 USA

**Keywords:** Community ecology, Ecosystem ecology, Freshwater ecology, Population dynamics

## Abstract

Intraspecific interactions within predator populations can affect predator–prey dynamics and community structure, highlighting the need to better understand how these interactions respond to anthropogenic change. To this end, we used a half-century (1969–2018) of abundance and size-at-age data from Lake Erie’s walleye (*Sander vitreus*) population to determine how anthropogenic alterations have influenced intraspecific interactions. Before the 1980s, the length-at-age of younger walleye (ages 1 and 2) negatively correlated with older (age 3 +) walleye abundance, signaling a ‘density feedback’ in which intraspecific competition limited growth. However, after the early 1980s this signal of intraspecific competition disappeared. This decoupling of the density feedback was related to multiple anthropogenic changes, including a larger walleye population resulting from better fisheries management, planned nutrient reductions to improve water quality and transparency, warmer water temperatures, and the proliferation of a non-native fish with novel traits (white perch, *Morone americana*). We argue that these changes may have reduced competitive interactions by reducing the spatial overlap between older and younger walleye and by introducing novel prey. Our findings illustrate the potential for anthropogenic change to diminish density dependent intraspecific interactions within top predator populations, which has important ramifications for predicting predator dynamics and managing natural resources.

## Introduction

Apex predators have been severely impacted by anthropogenic environmental change^1,2^, which can dramatically alter both interspecific and intraspecific interactions^3^. Of particular concern are anthropogenic changes to predator ‘density feedbacks’^4^. These feedbacks are intraspecific interactions that intensify as conspecific density increases, such as competition, aggression, and cannibalism, with subsequent effects on predator population growth or factors that affect growth^4^. For example, density feedbacks have been shown to determine body size in fishes^5^, fecundity in birds^6^, mortality in mammals^7^, and the dispersal of predators into new habitats^8^. Through these influences on predator growth, reproduction, mortality, and dispersal, density feedbacks can in turn mediate food webs and community structure^9,10^ and the ecosystem services generated by predator populations (e.g., wildlife harvest^11^).

**Figure 1:**
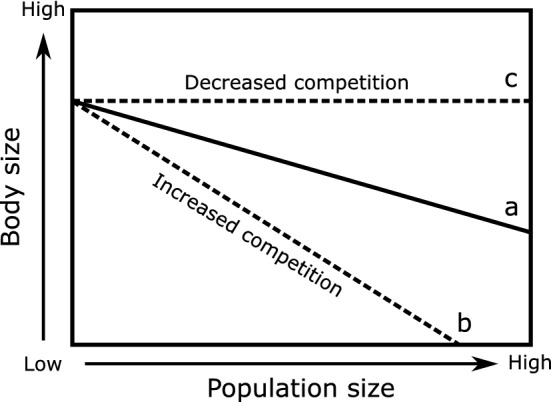
Predictions of how changes in the strength of intraspecific interactions could affect the relationship between predator growth (represented as body size) and population size. In (**a**), predator length declines as its population size increases owing to increased intraspecific interactions, such as competition or aggression, thus reducing the energy available for surplus growth. Environmental changes that (**b**) increase the frequency or intensity of intraspecific interactions would be expected to increase the slope of the relationship between body size and population size, with the opposite occurring if (**c**) the frequency or intensity of intraspecific interactions are reduced.

The nature of density feedbacks are often assumed to be fixed within a species (e.g., determined by life-history characteristics^11,12^), with environmental change simply altering the number of individuals or the carrying capacity producing a predictable response in the population^9,12,13^. However, human-induced environmental change has the potential to alter the intensity of intraspecific interactions such that population growth (or body size, fecundity, mortality, etc.) may no longer respond as expected to changes in population size^14,15^ (Fig. 1). For instance, the intensity of intraspecific interactions within a predator population can vary along environmental gradients, with stronger interactions occurring in areas with lower prey availability or less available habitat^16,17^. Anthropogenic stressors that create similar environmental changes, such as habitat fragmentation that crowds the same number of predators into a smaller area^18^, could therefore produce stronger than expected density feedbacks (Fig. 1, line b). Conversely, anthropogenic changes that reduce intraspecific interactions, such as via resource subsidies or the introduction of new prey^19,20^, could result in weaker than expected density feedbacks (Fig. 1, line c). This potential for anthropogenic alteration to density feedbacks, and the important ecological role played by predators, therefore necessitate a better understanding of how humans can affect the strength of predator density feedbacks.

This need is particularly pressing in large freshwater ecosystems, such as Lake Erie (USA-Canada), which are hotspots for anthropogenic environmental change^21^ and where competition within top predator populations affects the management of key ecosystem services. For example, walleye (*Sander vitreus*) in Lake Erie support one of the world’s largest freshwater fisheries^22^. The number and size of walleye available to this fishery have been historically affected by an age-structured density feedback. Specifically, during the 1930s–1970s, harvest-driven declines in the abundance of older walleye (age 3 +) tended to be matched by compensatory increases in the lengths^23^ and weights^24^ of younger walleye (age-1 and age-2 individuals), along with more rapid maturation^23^, higher fecundity^25^, and improved recruitment^26^. This compensatory relationship occurred because walleye growth has historically been prey-limited^26^ and both older and younger walleye compete for the same prey (specifically fishes, hereafter ‘prey’). Therefore, declines in the abundance of older walleye, whether due to exploitation or natural dynamics, improved the growth of younger walleye by reducing competition for prey^23,27^.

**Figure 2:**
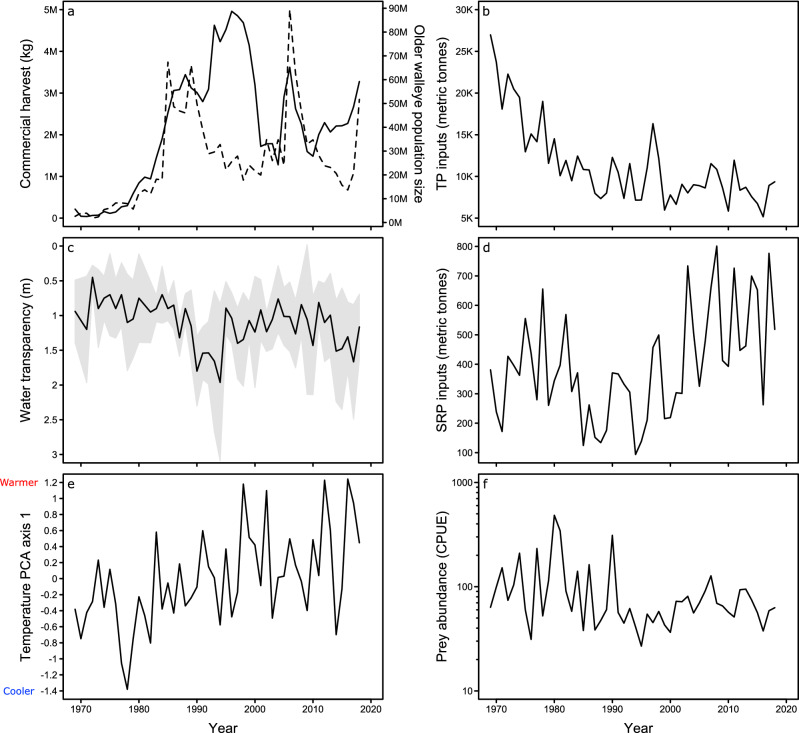
Anthropogenic changes in Lake Erie during 1969–2018. Changes include: (**a**) total lakewide commercial walleye harvest (solid line); (**b**) annual lakewide total phosphorus (TP) inputs; (**c**) mean annual water transparency in the western basin (gray shading indicates the standard deviation); (**d**) total annual soluble reactive phosphorus (SRP) inputs from the Maumee River; (**e**) climate variability, represented using an ordination axis of seasonal air and water temperatures (Supplementary Information – Sect. 2); and (**f**) total prey abundance (CPUE; individuals·trawl min^−1^). We also plotted (**a**) the estimated size of the older (age 3 +) walleye population (dashed line) to illustrate its relationship to commercial harvest.

However, anthropogenic changes to Lake Erie’s physical and biological environment may have altered this expected relationship. These changes include management actions during the 1970s, particularly a fishing moratorium that coincided with several large year-classes to greatly augment the size of the walleye population from a few million to consistently over 20 million by the mid-1980s^22^ (Fig. 2a, dashed line). Additionally, declines and then increases in anthropogenic nutrient inputs during the 1970s to present (Fig. 2b,c,d), and climate warming (Fig. 2e), have led to complex alterations in the availability of suitable thermo-optical habitat for both walleye and their prey^28^. Furthermore, the composition of Lake Erie’s prey community has changed^29^ due partly to the proliferation of two invasive fishes, the white perch (*Morone americana*) and the round goby (*Neogobius melanostomus*). The influence of these various anthropogenic perturbations on walleye depends on how they have affected intraspecific competition for prey. Competition, and thus the density feedback, may have intensified (Fig. 1, line b) if walleye or their prey have been compressed into a smaller area owing to reduced habitat availability from water warming and nutrient-driven hypolimnetic hypoxia^30^. Similarly, the expansion of non-native prey, which tend to be less preferred by walleye (see Supplementary Information–Sect. 1, Table S1.1), may have increased competition for the preferred prey that remain^31^. Conversely, the feedback may have weakened (Fig. 1, line c) if prey have become easier to obtain owing to improved walleye foraging efficiency (via nutrient-driven shifts in water transparency^32^) and if introduced prey have unique traits that provide a novel resource^33,34^. Consequently, walleye in Lake Erie provide an excellent system for investigating anthropogenic impacts on density feedbacks.

Here, we used a 50-year (1969–2018) dataset to first quantify anthropogenic changes in commercial harvest, nutrient inputs, water transparency, temperatures, and the prey community of Lake Erie. Next, we determined whether anthropogenic changes to the lake environment, prey abundances, or prey community (both species and traits) were related to a shift in the relationship between the body size of younger walleye and the abundance of older walleye (i.e., the density feedback).

## Results

### Anthropogenic environmental change

**Figure 3:**
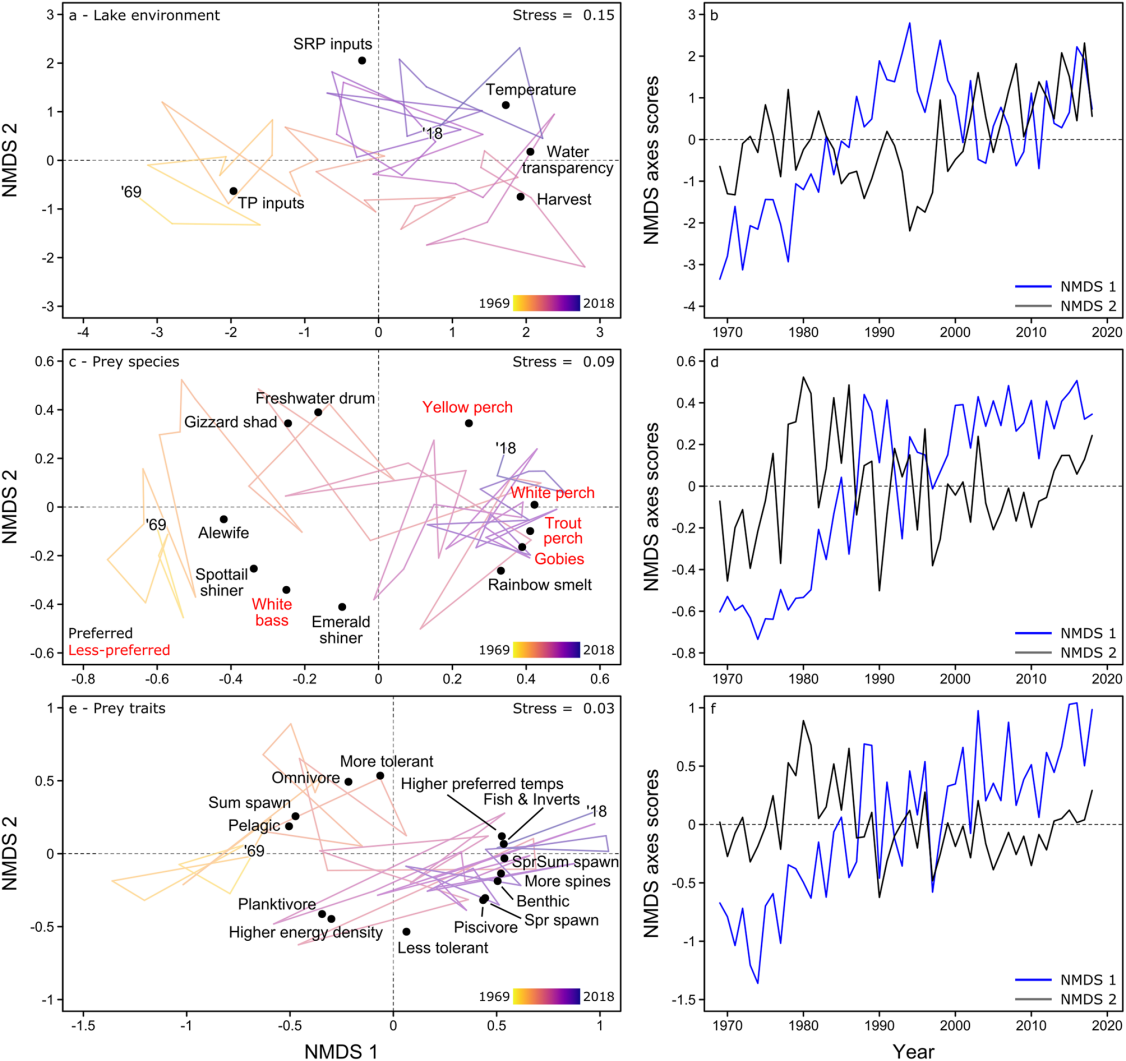
Ordinations portraying temporal shifts in Lake Erie’s (**a**, **b**) environment, (**c**, **d**) prey- species composition, and (**e**, **f**) prey-trait composition during 1969–2018 (‘69–‘18). Years in (**a**, **c**, **e**) progress temporally across a color gradient from yellow to blue, with orange and purple representing intermediate decades. In (**c**), text color indicates which prey are preferred (black text) versus less-preferred (red text) by Lake Erie walleye. Relationships between time and NMDS axis 1 (blue lines) and axis 2 (black lines) are illustrated in (**b**, **d**, **f**) to show which years are associated with large directional shifts in the ordinations.

The largest anthropogenic environmental changes occurred during the 1980s when total phosphorus (TP) inputs declined and commercial walleye harvest (also reflecting increased walleye abundances), water transparency, and temperature increased (moving from left to right primarily on NMDS axis 1 in Fig. 3a,b). Based on mean values before versus after the 1980s, TP inputs decreased from about 18,500 to 9100 metric tonnes (Fig. 2b), whereas commercial harvest increased from about 0.2 to 2.9 million kg (Fig. 2a), and water transparency increased from about 0.9 to 1.2 m (Fig. 2c; see Supplementary Information–Sect. 2, Fig. S2.1 for changes in seasonal temperatures). We also observed increases in soluble reactive phosphorus (SRP) inputs during the 2000s, which were primarily represented by the second ordination axis (moving from bottom to top on NMDS axis 2 in Fig. 3a). Before the 2000s (1969–1999), mean SRP inputs from the Maumee River were about 320 metric tonnes, which then increased to about 550 metric tonnes during 2010–2018 (Fig. 2d).

### Prey community change

The largest changes in prey-species composition began during the 1980s and further intensified after the 2000s (Fig. 3c,d). Before the 1980s (left side of NMDS axis 1 in Fig. 3c), when TP inputs were higher and commercial harvest, water transparency, and temperatures were lower, the prey community was characterized by higher abundances of prey species preferentially selected by walleye, such as emerald and spottail shiners (*Notropis atherinoides* and *N. hudsonius*), gizzard shad (*Dorosoma cepedianum*), and alewife (*Alosa pseudoharengus*; see Supplementary Information–Sect. 1, Table S1.1). After the 1980s, as TP inputs declined and commercial harvest, water transparency, and temperature increased, the prey community shifted towards dominance by less-preferred species, particularly invasive white perch (moving rightward primarily on NMDS axis 1 in Fig. 3c). This compositional change further intensified after the 2000s because composition only remained on the right side of NMDS axis 1 after this period (Fig. 3c,d). The second NMDS axis primarily represented more sporadic interannual shifts between specific species, such as years with higher abundances of freshwater drum (*Aplodinotus grunniens*) and gizzard shad versus higher abundances of emerald shiner and white bass (*Morone chrysops*; Fig. 3c).

Directional changes in prey-species composition were matched by coincident shifts in their trait composition, which was also primarily represented by NMDS axis 1 (Fig. 3e,f). Before the 1980s, the prey community was characterized by species that tend to reside in the water column, that are soft-rayed (i.e., no dorsal spines), that prefer cooler temperatures, and that spawn in summer (Fig. 3e). These species also tend to be planktivores or omnivores with a higher energy density. After the 1980s, community trait composition shifted towards species with an opposite set of traits. This shift intensified after the 2000s and has persisted up to 2018 (Fig. 3f). This newer prey assemblage was characterized by benthic, spiny-rayed species that prefer warmer temperatures and spawn earlier in the year (spring or spring/summer; Fig. 3e). These species also tend to be piscivores and invertivores with lower energy densities.

### Changes in the walleye density feedback

**Figure 4:**
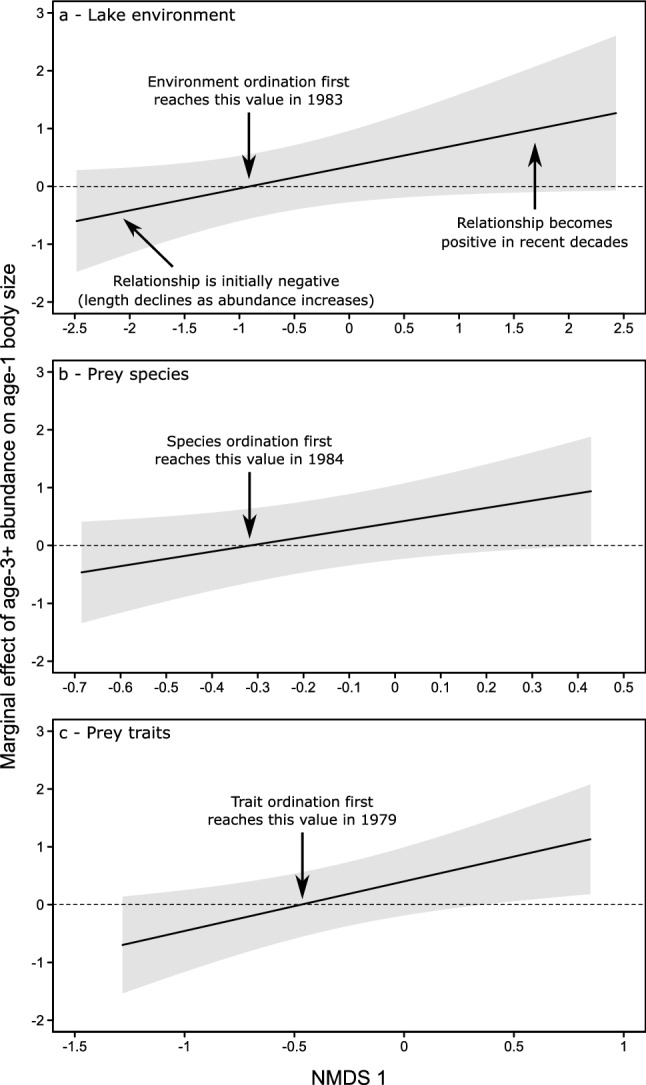
The marginal effects of older walleye (age 3 +) abundance on younger (age-1) walleye body size conditioned on changes in (**a**) the lake environment, (**b**) prey-species composition, and (**c**) prey-trait composition. Negative marginal effects indicate that the slope of the body size ~ abundance relationship is negative, specifically that younger walleye lengths tend to decline as older walleye abundance increases. A shift to a neutral then positive marginal effect indicates an inflection point where the slope changes and lengths no longer decline as abundance increases. Black lines are the estimated slope values extracted from the Generalized Least Squares models across the full range of NMDS axis values. Gray shaded areas indicate the confidence intervals of these estimates. Arrows are included in each panel at the inflection points along with which year the slopes first switched from negative to positive based on the corresponding years from the NMDS axes in Fig. 3b,d,f.

**Figure 5:**
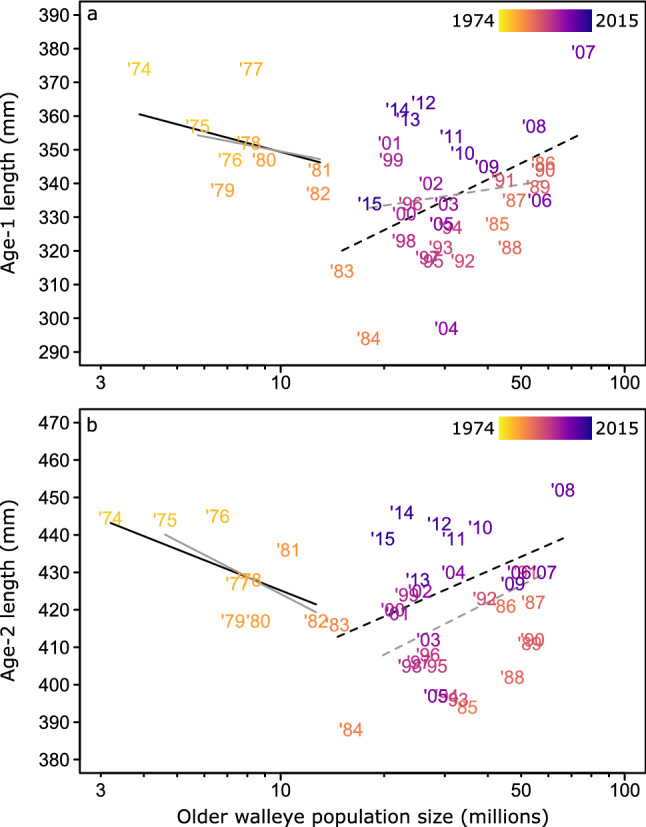
Relationships between the mean total length of (**a**) age-1 and (**b**) age-2 walleye and the estimated total number of older (age 3 +) walleye in Lake Erie during 1974–2015. Years progress across a color gradient from yellow to blue, with orange and purple representing intermediate decades. Solid lines indicate the best pre-breakpoint relationships, whereas dashed lines represent post-breakpoint relationships (modeled using piecewise linear regression). Gray lines show relationships with a downweighted influence of potentially high leverage points (age-1: 1974, 1983, 1984, and 2007; age-2: 1974, 1983, 1984, and 2008). Relationships were initially negative (age-1: slope =  − 51.2, R^2^_lik_ = 0.37, *P* = 0.041; age-2: slope =  − 42.0, R^2^_lik_ = 0.36, *P* = 0.046), but switched to positive after the breakpoint (age-1: slope = 52.2, R^2^_lik_ = 0.13, *P* = 0.028; age-2: slope = 46.4, R^2^_lik_ = 0.12, *P* = 0.042). Note the log-scale on the x-axis.

The slope of the density feedback shifted from negative to positive in both age-1 and age-2 walleye (Figs. 4 and 5 and Supplementary Information–Sect. 3). This shift was related to changes in the lake environment, prey-species composition, and prey-trait composition for age-1 walleye (based on significant interactions with the respective first NMDS axes; LRTs, *n* = 42; Environment: *L* = 4.08, df = 1, R^2^_lik_ = 0.092, *P* = 0.043; Species: *L* = 5.21, df = 1, R^2^_lik_ = 0.12, *P* = 0.022; Traits: *L* = 7.10, df = 1, R^2^_lik_ = 0.16, *P* = 0.0077; Fig. 4) and in relation to only prey-species composition for age-2 walleye (based on a significant interaction with NMDS axis 1; LRT, *n* = 42, *L* = 5.67, df = 1, R^2^_lik_ = 0.13, *P* = 0.017; Fig. S3.1). We found no significant changes in relation to prey abundance, nor with any of the second NMDS axes.

Based on the conditional marginal effects, the slope of the age-1 density feedback shifted from negative to positive around a value of -0.9 on NMDS axis 1 of the environmental ordination (Fig. 4a), around -0.3 on NMDS axis 1 of the prey-species ordination (Fig. 4b), and around -0.5 on NMDS axis 1 of the prey-trait ordination (Fig. 4c). These values corresponded to changes during the late 1970s to mid-1980s in aspects of the lake environment (specifically TP, harvest, temperature, and water transparency) and prey (e.g., losses of alewife and shiners versus gains in white perch), which were primarily associated with NMDS axis 1 in their respective ordinations. Similarly, the slope of the age-2 density feedback also shifted from negative to positive around a value of -0.6 on NMDS axis 1 of the prey-species ordination (Fig. S2), which corresponded to shifts in prey-species composition during the late 1970s to early 1980s (Fig. 3d).

Our breakpoint analyses further supported the early 1980s as the timing of major changes in the density feedback. Based on the mid-1980s as the likely timing of the shift in the age-1 and age-2 density feedbacks, we selected 1980 through 1990 as the candidate years for our breakpoint analyses. These models then indicated that 1982 was the best-supported breakpoint year for the shift in the density feedback for both age-1 and age-2 walleye (Fig. 5).

## Discussion

Our long-term (1969–2018) analyses of the Lake Erie ecosystem indicated that a shift occurred in the slope of the walleye density feedback, from negative to positive, likely during the early 1980s. The nature of this shift indicated a decoupling of the dependence of younger walleye lengths on older walleye abundance, signaling a reduction in the intensity of intraspecific competition. This shift was related to human-driven changes in commercial walleye harvest, nutrient inputs (primarily TP), water transparency, and temperature, in addition to a shift in both prey-species and prey-trait composition. These findings raise two key questions: (1) Why did the signal of intraspecific competition disappear? and (2) What mechanisms generated the recent positive relationship?

One explanation for the disappearance of the density feedback in the early 1980s is that anthropogenic environmental change reduced competition by creating greater spatial separation between younger and older walleye. All age-classes of walleye generally congregate in the western basin, and thus interact, during the spring spawning season^22^. After spawning, many older walleye migrate to the central and eastern basins or other connected lakes (e.g., Lake Huron) where water temperatures are cooler and the abundance of preferred prey is higher^22^. The extent of these migrations may have increased owing to multiple abiotic and biotic drivers. One potential driver is population size. Walleye migrations into other basins and nearby lakes, such as Lake Huron, can be more extensive during high abundance years (e.g., 2008–2009^35^) versus low abundance years (e.g., 2011–2014^36^). The dramatic anthropogenic increase in the size of the walleye population during the 1980s (also reflected by an increase in commercial harvest) may therefore have driven more extensive migrations of older walleye out of the western basin. Lake Erie has also been warming since at least the 1960s^37^ and a warmer western basin may be causing older walleye to leave earlier in the season^38^. This inference is supported by reports of earlier migrations of older walleye in recent decades, with migrations mostly finishing by June during the 2010s^39^ compared to July during the 1990s^40^, suggesting that migrations may have been occurring progressively earlier as the lake warmed. Additionally, declines in TP inputs during the 1980s may have furthered these migrations by improving hypolimnetic dissolved oxygen conditions and thus habitat availability in the central and eastern basins^41,42^. However, reduced nutrient inputs have likely played a smaller role compared to population size and temperature given that nutrient inputs and its impairments have increased in Lake Erie since the mid-1990s^43^ without an associated change in the density feedback. Ultimately, although we hypothesize that the shift in the density feedback could be driven by changing walleye migrations, we cannot confirm this possibility due to a lack of historical walleye movement data.

Changes in the prey community may also have contributed to walleye movements and thus a reduction in intraspecific competition. Older walleye migrate not only to seek out better thermal habitat but also in pursuit of preferred prey^39,40^. The replacement of preferred prey in the western basin (i.e., pelagic, soft-rayed, and higher energy species such as shiners and alewife^44^) with less-preferred species (i.e., benthic, spiny-rayed, lower energy invaders like white perch) could therefore contribute to earlier or more extensive migrations of older walleye in pursuit of better prey opportunities. This shift in prey composition was likely driven by several anthropogenic changes to the ecosystem. First, declines in nutrient inputs owing to phosphorus abatement programs^41^ have been associated with reduced zooplankton biomass^45^ and thus losses of zooplanktivorous prey preferred by walleye^27,44^. Second, better fishery and nutrient management have respectively led to increased walleye abundance^22^ and water transparency^41^, which contributed to declines in preferred prey by increasing predation pressure^44,46^. Finally, invasive white perch expanded dramatically during the early 1980s owing to reduced competition (via declines in other prey species^47^) and climate warming that reduced the mortality of white perch during cold winters^48^. Collectively, this combination of planned and unplanned anthropogenic changes likely drove the shift from preferred to less-preferred prey in Lake Erie.

An alternative (or additional) explanation for the shift in the density feedback is that the increase in white perch provided a new prey resource for walleye, thus reducing intraspecific competition. The timing of the initial shift in the density feedback around the early 1980s coincides closely with the expansion of invasive white perch in Lake Erie^49^. Our dataset first records this species in 1977 and its abundance increased by an order of magnitude in 1980 and again in 1984 and it has dominated the western basin prey community ever since (on average comprising over 50% of total prey abundance annually). Other notable Lake Erie invaders, such as round goby and dreissenid mussels, did not proliferate until after the early 1990s. It may seem counterintuitive for white perch to reduce intraspecific competition for prey given that total prey abundance has not changed (see Fig. 2f) and white perch is both well-defended by its dorsal spines and has one of the lowest energy densities of the prey community (Supplementary Information – Sect. 4). However, even well-defended, low energy prey can be beneficial for walleye, which is an opportunistic generalist that can rapidly adjust to shifts in prey composition^46,50^ and can feed and grow well on prey communities dominated by less-preferred species^51^. Additionally, white perch may provide a novel resource by occupying a different temporal and spatial niche compared to walleye’s preferred prey, which could augment overall prey availability^33,52^. For instance, walleye and white perch both spawn during spring, whereas walleye’s preferred prey typically spawn during summer. The early life stages of white perch may therefore fill a temporal gap in prey availability for walleye. In support of this assertion, walleye diets in Lake Erie can include large quantities of white perch larvae^27^ and walleye in other lakes prey heavily on white perch during spring until preferred prey become more abundant later in the year^53^. White perch also occupy habitats lower in the water column (benthopelagic or benthic) compared to the primarily pelagic preferred prey. Invasions of other benthic fishes have increased prey availability for walleye (e.g., round goby^34,50^) and we suspect the same could be true for white perch. This novel benthic resource may therefore have reduced intraspecific competition for pelagic prey, thus contributing to the decoupling of the density feedback.

We cannot fully explain the emergence of the positive relationship between older walleye abundance and younger walleye lengths in recent decades, even after conducting several post-hoc analyses (detailed below). For example, we explored the possibility that the relationship is neutral and only appears positive because it is driven by a few extreme values in certain years, specifically 1974, 1983, 1984, 2007, and 2008. However, downweighting the influence of these potentially high leverage years did not alter our results (Fig. 5). We also investigated the possibility that intraspecific competition now affects aspects of younger walleye other than length, such as weight or maturation. While we only have data on these factors starting in the 1980s, we again found no indication of a negative relationship between these metrics and older walleye abundance (Supplementary Information – Sect. 5), providing further evidence that intraspecific competition truly has relaxed (although note that competition within age classes is still important^54^). Finally, if length is no longer influenced by intraspecific competition between younger and older individuals, then a positive relationship could result if both metrics now respond in the same way to another component of the ecosystem. Prey availability is one possibility because high prey abundance can enhance both growth and survival^26^. We found support for this explanation in a shift to a more positive relationship between younger walleye lengths and prey abundance after 1982 (Supplementary Information – Sect. 6, Fig. S6.1), which matched the estimated timing of the shift in the density feedback. This finding suggests that younger walleye lengths may now be more related to prey availability rather than intraspecific competition with older individuals. Additionally, environmental factors may be exerting stronger control compared to earlier decades now that the influence of intraspecific competition has weakened. For instance, walleye lengths during recent decades are related to lake water levels^54^, which can determine the availability and quality of littoral spawning habitat^55^. Because high-quality spawning conditions can also improve walleye recruitment^56^, years with suitable water levels could translate to both larger young walleye and higher abundances of older individuals.

Our results have important implications for both basic and applied science. From a basic science perspective, our results provide evidence that density feedbacks within a species may be more plastic than expected^14,15^ and can shift in ecologically surprising ways in response to anthropogenic environmental change (i.e., changing unpredictably or counterintuitively^57^). We consider our results surprising because Lake Erie’s walleye population exhibited a signal of intraspecific competition between older and younger walleye during the 1970s, whereas during the 1980s when the population became an order of magnitude larger, competition should have intensified but instead it diminished. Walleye also now counterintuitively seem to be larger during years with more individuals in the lake. While we can only speculate on the exact mechanism(s) underlying this finding, it provides novel evidence that expected intraspecific interactions can weaken in response to anthropogenic change and the nature of established relationships can even be reversed. Similar ecological surprises are emerging from novel terrestrial and aquatic ecosystems worldwide^57^, suggesting that our example may just be one of many counterintuitive changes that have occurred in predator populations. Such changes must be identified and studied to predict future changes in predator population dynamics, predator–prey interactions, and associated communities.

From an applied science perspective, our results also show the need for resource managers to periodically revisit assumptions made regarding density feedbacks in managed populations. Harvest allocation strategies for maintaining wildlife populations at sustainable levels often involve population models that incorporate density feedbacks^11,58^. Such models frequently assume that increasing harvest in years of high population abundance will benefit population growth by reducing intraspecific interactions with younger individuals. For example, in Lake Erie, allowing for high walleye harvest in years of high population abundance has been suggested to be beneficial to walleye growth and recruitment to the fishery because it improves resource availability for younger walleye^27^. However, harvesting more older individuals may now have little effect on the growth of younger individuals given that the density feedback no longer exists. Sudden shifts in the dynamics of key species can also be indicative of broader population- or ecosystem-level changes that may need to be incorporated into management efforts^59,60^. Continuing with Lake Erie as an example, the disappearance of the density feedback is itself indicative of broader changes in the environment and prey community, including potential changes in walleye movements and their impacts on the food web (e.g., feeding elsewhere and supplementing their diet with an invasive species). These examples demonstrate how management strategies may need to be altered, or new strategies developed, to account for anthropogenic changes in expected density feedbacks and the impacts of these changes on the broader ecosystem. Only by monitoring and periodically re-evaluating the relationship between predator growth and predator population size can we develop appropriate strategies to prevent detrimental outcomes, sustainably manage exploited populations, and conserve threatened ones.

## Methods

We focused our analyses on Lake Erie’s western basin, which is where younger and older walleye generally interact during spring when the older individuals aggregate for spawning^22^. During summer and fall, older walleye tend to migrate eastwards into the lake’s deeper central and eastern basins in pursuit of cooler temperatures and more abundant prey, whereas the younger individuals tend to remain in the western basin^22^.

### Lake Erie environment

We collected data on four aspects of the lake environment that have been impacted by humans and that can affect walleye and their intraspecific interactions: commercial harvest, nutrient availability, water transparency, and temperature.

Commercial harvest – We quantified walleye harvest levels using lakewide data on the annual total commercial walleye harvest (kg) from US and Canadian waters during 1969–2018 from the Great Lakes Fishery Commission^61^. Most of this harvest has occurred on the Canadian side since the closure of the US commercial walleye fishery in 1970^22^. Commercial harvest reflects both the number of walleye removed from Lake Erie and (to a degree) long-term anthropogenic trends in walleye population size given that harvest tended to be low during the 1970s when the walleye population was smaller and higher during the 1980s and onwards as the population recovered (Fig. 2a).

Nutrient availability and water transparency – We quantified nutrient availability using annual inputs of total phosphorus (TP; metric tonnes) during 1969–2018^62^ (sourced from www.blueaccounting.org). To capture changes in more bioavailable forms of phosphorus, we also collected data on annual inputs of soluble reactive phosphorus (SRP; metric tonnes) from the Maumee River during 1975–1978 and 1982–2018 (source: www.ncwqr.org/monitoring). Although SRP data were not available for the unmonitored years (specifically 1969–1974 and 1979–1981), SRP is positively related to river discharge^63^ and river discharge data were available. Therefore, we estimated SRP for the unmonitored years based on a linear regression of SRP inputs to river discharge during 1975–1995 (R^2^ = 0.44; *P* = 0.002).

Because walleye is a visual predator and prey use low light levels as a predation refuge^32^, we also included an index of water transparency (Secchi depth to the nearest 10 cm) to account for nutrient-driven changes in the light environment^41^. Measurements were collected at six sites in the western basin by the Ohio Department of Natural Resources-Division of Wildlife (ODNR-DOW) during fall 1969–2018 and averaged to produce annual estimates (Supplementary Information – Sect. 1). Fall Secchi depth is the most complete water transparency dataset available for Lake Erie and reflects long-term trends in the light environment caused by changes in phosphorus inputs^41^.

Temperature – We represented changes in Lake Erie temperatures using a combination of: (i) modeled monthly mean surface water temperatures during 1969–2018, obtained from the National Oceanic and Atmospheric Administration (NOAA) Great Lakes Seasonal Hydrological Forecasting System; and (ii) daily mean minimum observed air temperatures during 1969–2018 measured at the Toledo, OH airport (www.ncdc.noaa.gov), which were highly correlated to maximum temperatures and were included as observed data to support modeled water temperatures. We converted monthly and daily values to annual seasonal means by averaging temperatures for winter (December–February), spring (March–May), summer (June–August), and fall (September–November) in each year and then converted these variables to *z*-scores by centering them to their respective means and dividing by their standard deviations. We then used Principal Component Analysis (PCA) to decompose the scaled seasonal surface water and air temperatures into their principal axes of variation and extracted the first PCA axis for our analyses because it represents most of the annual variability in temperature (59.8%; Supplementary Information – Sect. 2).

### Prey community

To quantify prey abundance and composition, we used annual catch-per-unit-effort (CPUE; individuals·min^-1^) data from daytime bottom trawls conducted by the ODNR-DOW in the western basin in fall (mid-September through mid-October) during 1969–2018. We calculated annual prey-species composition based on the abundances of 11 species common in walleye diets (Supplementary Information – Sect. 1, Table S1.1). We also collated data on prey functional traits because intraspecific interactions among walleye may respond more strongly to changes in prey traits rather than species. We selected traits that might respond to environmental changes in Lake Erie or that are relevant to interactions with walleye. These traits included physiological tolerances to anthropogenic stress, habitat and feeding preferences, defense mechanisms, and prey energy density. All trait values were obtained from existing databases and the primary literature (Supplementary Information – Sect. 4).

### Walleye body size and abundance

Annual length-at-age data for younger walleye (age-1 and age-2) were available during 1974–2015 from ODNR-DOW gillnet surveys conducted during fall typically after thermal stratification has ended and when walleye have accomplished most of their growth for the year^27^. The number of individuals caught in these surveys ranged from 148 to 4268 depending on the size of the spawning walleye population, with an annual average of about 1300 ± 100 individuals (mean ± SD). However, no age-1 walleye were caught in 2003 owing to low reproduction so we predicted the mean length for this year based on a positive linear relationship to the mean length of the age-2 population in the following year (R^2^ = 0.64; *P* < 0.001).

The abundances of older (age 3 +) walleye during 1969–2018 were based on lakewide annual estimates of the total size of the age 3 + population obtained from statistical catch-at-age model outputs produced by the Lake Erie Committee’s Walleye Task Group^64^. Statistical catch-at-age models are a type of integrated population model, which incorporate multiple data sources, including fishery-dependent and fishery-independent data, into a single analysis to estimate population demographics and dynamics^65^. Two different statistical catch-at-age models have been used to produce the estimates of the size of the adult walleye population: an initial catch-at-age model used during 1969–1999 and a modern version using data from 1978 to present^64,66^. To create a single time-series for 1969–2018, we combined the 1969–1977 estimates with the 1978–2018 estimates and further corrected the initial catch-at-age values based on a linear regression of the overlapping years between both models (1978–1999; R^2^ = 0.95, *P* < 0.001). Note that no live fishes were used in this study (only data) and all fish collections by the ODNR-DOW were conducted in adherence to the American Fisheries Society's "Guidelines for the Use of Fishes in Research" (https://fisheries.org/policy-media/science-guidelines/guidelines-for-the-use-of-fishes-in-research/).

### Statistical analyses

To quantify anthropogenic changes in the lake, we decomposed the environmental variables, prey taxonomic composition, and prey trait composition into their principal axes of variation using Non-metric Multidimensional Scaling (NMDS) with two-dimensional solutions performed via the ‘vegan’ package in R^67^. Before this analysis, the environmental variables were converted to *z*-scores, prey-species composition was converted to Hellinger-transformed abundances, and prey-trait composition was converted to community-weighted mean trait values^68^.

To test for changes in the density feedback, we used eight Generalized Least Squares (GLS) regression models (performed via the ‘nlme’ package in R^69^) to determine whether the slope of the relationship between younger walleye body size and older walleye abundance changed in relation to changes in the lake environment or prey community. We used separate models because age-1 and age-2 walleye may respond differently to competition with older walleye^70^, and because we found that changes in the lake environment and prey community co-occurred and were thus correlated (see Fig. 3) so they could not be examined in the same models. All models related either the mean annual length of age-1 (four models) or age-2 (four models) walleye to the annual abundance of older walleye, which was also log-transformed to improve linearity. To test for changes in the slope of this relationship, we then included interactions with the annual values for either the lake environment (two NMDS axes), total prey abundance (summed CPUE across all species; Fig. 2f), prey-species composition (two NMDS axes), or prey-trait composition (two NMDS axes). Annual values do not fully represent the conditions that younger walleye have experienced across their two (age-1) or three (age-2) years of life, thus we also averaged all predictors across each year and the previous year for the age-1 models and each year and the previous two years for the age-2 models. Each model also included a predictor of the annual number of growing degree days during the gill net survey to control for interannual differences in survey duration and a first-order autoregressive structure to control for temporal autocorrelation (Supplementary Information – Sect. 5). All predictors were converted to *z*-scores prior to modeling. Significant (*P* < 0.05) interactions were determined by removing them from the model and comparing the fuller versus reduced models using Likelihood Ratio Tests (LRTs) and maximum likelihood. We also report the partial R^2^ of these relationships (R^2^_lik_; calculated using the ‘R2.lik’ function from the ‘rr2’ package in R^71^), which is the difference in the variance explained between the model with and without the interaction based on maximum likelihood.

To visualize any detected interactions, we calculated the marginal effect of older walleye abundance on younger walleye body size conditioned on the effect of any interacting predictor (performed via the ‘marginaleffects’ package in R^72^). The result is a series of estimated slopes and their confidence intervals for the body size ~ abundance relationships extracted from each GLS model across the full range of values of any interacting NMDS axes. We also determined the approximate timing of any major shifts in these relationships based on which NMDS axis drove the interaction and the timing of change along this axis that corresponded to an inflection in the slope of the density feedback (e.g., shifting from negative to neutral). In summary, the GLS models tested whether the slope of the body size ~ abundance relationship changed in relation to the lake environment or prey community and the marginal effects analyses visualize the nature of these slope changes.

While the GLS and marginal effects analyses determined how the density feedback changed, we needed an additional analysis to verify the estimated timing of these changes. To do so, we conducted iterative piecewise regressions^73^ (also called segmented regression or ‘breakpoint’ analysis) of the relationships between older walleye abundance and age-1 and age-2 walleye body size. This method is used to identify where an inflection occurs in the relationship between *x* and *y*, with the iterative part referring to iteratively testing a set of candidate breakpoints to identify which breakpoint produces the best-fitting models. We used this analysis to fit models to earlier versus later data given that we found the body size ~ abundance relationship changed through time. We chose potential breakpoints of 1980–1990 based on the marginal effects analyses that suggested the relationship changed around the early 1980s. We fit new GLS models to the data from before and after these different candidate years and selected the breakpoint with the lowest mean squared error across models. Significance (*P* < 0.05) of breakpoint models was also determined using LRTs, with R^2^_lik_ for these models reflecting the variance explained by the term for older walleye abundance.

## Supplementary Information

Supplementary Information 1.

Supplementary Information 2.

## Data Availability

All data is publicly available from the Dryad Digital Repository at 10.5061/dryad.hqbzkh1mc.

## References

[1] Estes JA (2011). Trophic downgrading of planet Earth. Science.

[2] Wilson MW (2020). Ecological impacts of human-induced animal behaviour change. Ecol. Lett..

[3] Guiden PW, Bartel SL, Byer NW, Shipley AA, Orrock JL (2019). Predator–prey interactions in the Anthropocene: reconciling multiple aspects of novelty. Trends Ecol. Evol..

[4] Herrando-Pérez S, Delean S, Brook BW, Bradshaw CJA (2012). Density dependence: an ecological Tower of Babel. Oecologia.

[5] Byström P, García-Berthou E (1999). Density dependent growth and size specific competitive interactions in young fish. Oikos.

[6] Dhondt AA, Kempenaers B, Adriaensen F (1992). Density-dependent clutch size caused by habitat heterogeneity. J. Anim. Ecol..

[7] Cubaynes S (2014). Density-dependent intraspecific aggression regulates survival in northern Yellowstone wolves (Canis lupus). J. Anim. Ecol..

[8] Matthysen E (2005). Density-dependent dispersal in birds and mammals. Ecography.

[9] Sinclair ARE, Pech RP (1996). Density dependence, stochasticity, compensation and predator regulation. Oikos.

[10] Hauzy C, Gauduchon M, Hulot FD, Loreau M (2010). Density-dependent dispersal and relative dispersal affect the stability of predator–prey metacommunities. J. Theor. Biol..

[11] Rose KA, Cowan JH, Winemiller KO, Myers RA, Hilborn R (2001). Compensatory density dependence in fish populations: importance, controversy, understanding and prognosis. Fish Fish..

[12] Fowler CW (1981). Density dependence as related to life history strategy. Ecology.

[13] Reynolds SA, Brassil CE (2013). When can a single-species, density-dependent model capture the dynamics of a consumer-resource system?. J. Theor. Biol..

[14] Murray DL, Anderson MG, Steury TD (2010). Temporal shift in density dependence among North American breeding duck populations. Ecology.

[15] Borlestean, A., Frost, P. C. & Murray, D. L. A mechanistic analysis of density dependence in algal population dynamics. *Front. Ecol. Evol.* **3**, (2015).

[16] Wildy EL, Chivers DP, Kiesecker JM, Blaustein AR (2001). The effects of food level and conspecific density on biting and cannibalism in larval long-toed salamanders. Ambystoma macrodactylum. Oecologia.

[17] Pafilis P, Meiri S, Foufopoulos J, Valakos E (2009). Intraspecific competition and high food availability are associated with insular gigantism in a lizard. Naturwissenschaften.

[18] Metcalf CJE, Hampson K, Koons DN (2007). What happens if density increases? Conservation implications of population influx into refuges. Anim. Conserv..

[19] Svanbäck R, Bolnick DI (2005). Intraspecific competition affects the strength of individual specialization: an optimal diet theory method. Evol. Ecol. Res..

[20] Newsome TM (2015). The ecological effects of providing resource subsidies to predators. Glob. Ecol. Biogeogr..

[21] Strayer DL, Dudgeon D (2010). Freshwater biodiversity conservation: recent progress and future challenges. J. North Am. Benthol. Soc..

[22] Vandergoot, C. S. *et al.* Back from the Brink: Sustainable Management of the Lake Erie Walleye Fishery. in (eds. Krueger, C. C., Taylor, W. W. & Youn, S.) 431–466 (American Fisheries Society, 2019).

[23] Hatch RW, Nepszy SJ, Muth KM, Baker CT (1987). Dynamics of the recovery of the western Lake Erie walleye (Stizostedion vitreum vitreum) stock. Can. J. Fish. Aquat. Sci..

[24] Parsons JW (1970). Walleye fishery of Lake Erie in 1943–62 with emphasis on contributions of the 1942–61 year-classes. J. Fish. Board Can..

[25] Moles MD (2011). Reproductive divergence between growth forms of Lake Winnipeg walleye (Sander vitreus). Ecol. Freshw. Fish.

[26] Madenjian CP, Tyson JT, Knight RL, Kershner MW, Hansen MJ (1996). First-year growth, recruitment, and maturity of walleyes in western Lake Erie. Trans. Am. Fish. Soc..

[27] Hartman KJ, Margraf FJ (1992). Effects of prey and predator abundances on prey consumption and growth of walleyes in western Lake Erie. Trans. Am. Fish. Soc..

[28] Collingsworth PD (2017). Climate change as a long-term stressor for the fisheries of the Laurentian Great Lakes of North America. Rev. Fish Biol. Fish..

[29] Fraker ME, Sinclair JS, Frank KT, Hood JM, Ludsin SA (2022). Temporal scope influences ecosystem driver-response relationships: A case study of Lake Erie with implications for ecosystem-based management. Sci. Total Environ..

[30] Brandt SB (2011). Does hypoxia reduce habitat quality for Lake Erie walleye (Sander vitreus)? A bioenergetics perspective. Can. J. Fish. Aquat. Sci..

[31] Nelson DWM, Crossland MR, Shine R (2011). Foraging responses of predators to novel toxic prey: effects of predator learning and relative prey abundance. Oikos.

[32] Nieman CL, Gray SM (2019). Visual performance impaired by elevated sedimentary and algal turbidity in walleye Sander vitreus and emerald shiner Notropis atherinoides. J. Fish Biol..

[33] Dijkstra JA, Lambert WJ, Harris LG (2013). Introduced species provide a novel temporal resource that facilitates native predator population growth. Biol. Invasions.

[34] Johnson TB, Bunnell DB, Knight CT (2005). A potential new energy pathway in central Lake Erie: the round goby connection. J. Gt. Lakes Res..

[35] evidence from genetic stock identification (2015). Brenden, T. O. *et al.* Contributions of Lake Erie and Lake St. Clair walleye populations to the Saginaw Bay, Lake Huron, recreational fishery. North Am. J. Fish. Manag..

[36] Hayden TA (2019). Telemetry reveals limited exchange of walleye between Lake Erie and Lake Huron: Movement of two populations through the Huron-Erie corridor. J. Gt. Lakes Res..

[37] Beeton AM (1961). Environmental Changes in Lake Erie. Trans. Am. Fish. Soc..

[38] Dippold DA, Adams GD, Ludsin SA (2020). Spatial patterning of walleye recreational harvest in Lake Erie: Role of demographic and environmental factors. Fish. Res..

[39] Raby GD (2018). Does behavioural thermoregulation underlie seasonal movements in Lake Erie walleye?. Can. J. Fish. Aquat. Sci..

[40] Wang, H.-Y. *et al.* Movement of walleyes in Lakes Erie and St. Clair inferred from tag return and fisheries data. *Trans. Am. Fish. Soc.* **136**, 539–551 (2007).

[41] Ludsin SA, Kershner MW, Blocksom KA, Knight RL, Stein RA (2001). Life after death in Lake Erie: nutrient controls drive fish species richness, rehabilitation. Ecol. Appl..

[42] Sinclair JS (2021). Functional traits reveal the dominant drivers of long-term community change across a North American Great Lake. Glob. Change Biol..

[43] Scavia D (2014). Assessing and addressing the re-eutrophication of Lake Erie: Central basin hypoxia. J. Gt. Lakes Res..

[44] Knight RL, Vondracek B (1993). Changes in prey fish populations in western Lake Erie, 1969–88, as related to walleye, Stizostedion vitreum, predation. Can. J. Fish. Aquat. Sci..

[45] Conroy JD (2005). Temporal trends in Lake Erie plankton biomass: roles of external phosphorus loading and dreissenid mussels. J. Gt. Lakes Res..

[46] Knight RL, Margraf FJ, Carline RF (1984). Piscivory by walleyes and yellow perch in western Lake Erie. Trans. Am. Fish. Soc..

[47] Hartman KJ, Vondracek B, Parrish DL, Muth KM (1992). Diets of emerald and spottail shiners and potential interactions with other western Lake Erie planktivorous fishes. J. Gt. Lakes Res..

[48] Johnson TB, Evans DO (1990). Size-dependent winter mortality of young-of-the-year white perch: climate warming and invasion of the Laurentian Great Lakes. Trans. Am. Fish. Soc..

[49] Boileau MG (1985). The expansion of white perch, Morone americana, in the Lower Great Lakes. Fisheries.

[50] Pothoven SA, Madenjian CP, Höök TO (2017). Feeding ecology of the walleye (Percidae, Sander vitreus), a resurgent piscivore in Lake Huron (Laurentian Great Lakes) after shifts in the prey community. Ecol. Freshw. Fish.

[51] Kolar CS, Wahl DH, Hooe ML (2003). Piscivory in juvenile walleyes: relative importance of prey species, timing of spawning of prey fish, and density on growth and survival. Trans. Am. Fish. Soc..

[52] Cattau CE, Fletcher RJ, Reichert BE, Kitchens WM (2016). Counteracting effects of a non-native prey on the demography of a native predator culminate in positive population growth. Ecol. Appl..

[53] Gosch NJC, Pierce LL, Pope KL (2010). The effect of predation on stunted and nonstunted white perch. Ecol. Freshw. Fish.

[54] Gíslason D, McLaughlin RL, Robinson BW (2021). Synchronous changes in length at maturity across four species of Lake Erie fish with different harvest histories. Can. J. Fish. Aquat. Sci..

[55] Papenfuss JT, Cross T, Venturelli PA (2018). A comparison of the effects of water-level policies on the availability of walleye spawning habitat in a boreal reservoir. Lake Reserv. Manag..

[56] Raabe JK, VanDeHey JA, Zentner DL, Cross TK, Sass GG (2020). Walleye inland lake habitat: considerations for successful natural recruitment and stocking in North Central North America. Lake Reserv. Manag..

[57] Paine RT, Tegner MJ, Johnson EA (1998). Compounded perturbations yield ecological surprises. Ecosystems.

[58] Guthery FS, Shaw JH (2013). Density dependence: Applications in wildlife management. J. Wildl. Manag..

[59] Cerini F, Childs DZ, Clements CF (2023). A predictive timeline of wildlife population collapse. Nat. Ecol. Evol..

[60] Zhang F (2020). Early warning signals of population productivity regime shifts in global fisheries. Ecol. Indic..

[61] GLFC (Great Lakes Fishery Commission). Commercial fish production in the Great Lakes 1867–2020 [online database]. (2022).

[62] Maccoux MJ, Dove A, Backus SM, Dolan DM (2016). Total and soluble reactive phosphorus loadings to Lake Erie: a detailed accounting by year, basin, country, and tributary. J. Gt. Lakes Res..

[63] Baker DB (2019). Needed: early-term adjustments for Lake Erie phosphorus target loads to address western basin cyanobacterial blooms. J. Gt. Lakes Res..

[64] Wills, T. *et al. Report for 2019 by the Lake Erie walleye task group*. http://www.glfc.org/pubs/lake_committees/erie/WTG_docs/annual_reports/WTG_report_2020.pdf (2020).

[65] Maunder MN, Punt AE (2013). A review of integrated analysis in fisheries stock assessment. Fish. Res..

[66] Jones, M. L., Catalano, M. J. & Peterson, L. K. Stakeholder-centered development of a harvest control rule for Lake Erie walleye. in *Management Science in Fisheries* (Routledge, 2016).

[67] Oksanen, J. *et al.* vegan: Community ecology package. (2022).

[68] Lavorel S (2008). Assessing functional diversity in the field – methodology matters!. Funct. Ecol..

[69] Pinheiro, J., Bates, D., DebRoy, S., Sarkar, D. & R Core Team. nlme: Linear and nonlinear mixed effects models. (2020).

[70] Muth KM, Wolfert DR (1986). Changes in growth and maturity of walleyes associated with stock rehabilitation in western Lake Erie, 1964–1983. North Am. J. Fish. Manag..

[71] Ives AR, Li D (2018). `rr2`: An R package to calculate R^2^s for regression models. J. Open Source Softw..

[72] Arel-Bundock, V. marginaleffects: Marginal effects, marginal means, predictions, and contrasts. (2022).

[73] Bates, D. M. & Watts, D. G. *Nonlinear regression analysis and its applications*. (Wiley, 1988).

