# Supplementary material for: Anthropogenic change decouples a freshwater predator’s density feedback
Source: Sci Rep. 2023 May 10;13:7613. doi: 10.1038/s41598-023-34408-0 (PMC10172374; doi:10.1038/s41598-023-34408-0)
Supplement: Supplementary file 2 — Supplementary Information 2. [file 41598_2023_34408_MOESM2_ESM.docx]

**Title:** Anthropogenic change decouples a freshwater predator’s density feedback

**Authors:** Sinclair, J. S., Briland, R., Fraker, M. E., Hood, J. M., Frank, K. T., Faust, M. D., Knight, C., and Ludsin, S. A.

**Section 1 – Walleye and prey-fishes in Lake Erie**

Most walleye in Lake Erie are born during spring (typically March–April) in the western basin because it offers the greatest amount of suitable nursery habitat in the lake ^1^. Younger walleye (age-1 and age-2) tend to remain in the western basin, whereas many older walleye (age-3+) migrate to other basins and lakes during the summer and fall ^2^. Younger and older walleye therefore primarily overlap with one another (spatially and temporally) during the spawning season in the spring and early summer months in the western basin. Age-0 walleye initially feed on invertebrates but can quickly transition to piscivory within their first few months of life (e.g., ^3^). Afterwards, the diets of age-0 walleye include invertebrates and certain age-0 prey fishes (e.g., emerald shiner; ^4,5^), while age-1 and older walleye have similar diets comprised primarily of different prey-fish species (detailed in Table S1).

To estimate prey-fish abundances through time, we used data collected from bottom trawls conducted from September to October since 1969 in Lake Erie’s western basin by The Ohio Department of Natural Resources-Division of Wildlife (ODNR-DOW; Fig. S1.1). The trawls are designed to target percids, but also provide reliable estimates of the relative abundances of a variety of non-target species ^6^. All trawls were conducted during the daytime with a duration of 10–15 minutes using flat-bottom, semi-balloon otter trawls with a 10.7m headrope and a 6.4mm cod end mesh. Following collection, the catch from each trawl was sorted and counted, with subsampling (volumetric or gravimetric) used for extreme catches exceeding 1000 individuals. We also corrected for differences in sampling efficiency among vessels by using catch-per-unit-effort (CPUE) trawl values, which have been corrected by the ODNR-DOW for differences in fishing power across vessels and years ^6^.

**Table S1.1.** Common fish species consumed by age-1+ walleye in Lake Erie based on the diet contents of captured individuals ^4,5,7–10^. Prey species are sorted based on whether they are (‘Yes’) or are not (‘No’) preferentially selected for by walleye. We also list the age classes most vulnerable to walleye.

| **Common name** | **Scientific name** | **Preferred prey** | **Vulnerable age classes** |
| --- | --- | --- | --- |
| Alewife | *Alosa pseudoharengus* | Yes | Age 0 |
| Emerald shiner | *Notropis atherinoides* | Yes | All ages |
| Gizzard shad | *Dorosoma cepedianum* | Yes | Age 0 |
| Rainbow smelt | *Osmerus mordax* | Yes | All ages |
| Spottail shiner | *Notropis hudsonius* | Yes | All ages |
| Freshwater drum | *Aplodinotus grunniens* | No | Age 0 |
| Round goby | *Neogobius melanostomus* | No | All ages |
| Trout perch | *Percopsis omiscomaycus* | No | All ages |
| White bass | *Morone chrysops* | No | Age 0 |
| White perch | *Morone americana* | No | Age 0 |
| Yellow perch | *Perca flavescens* | No | Age 0 |


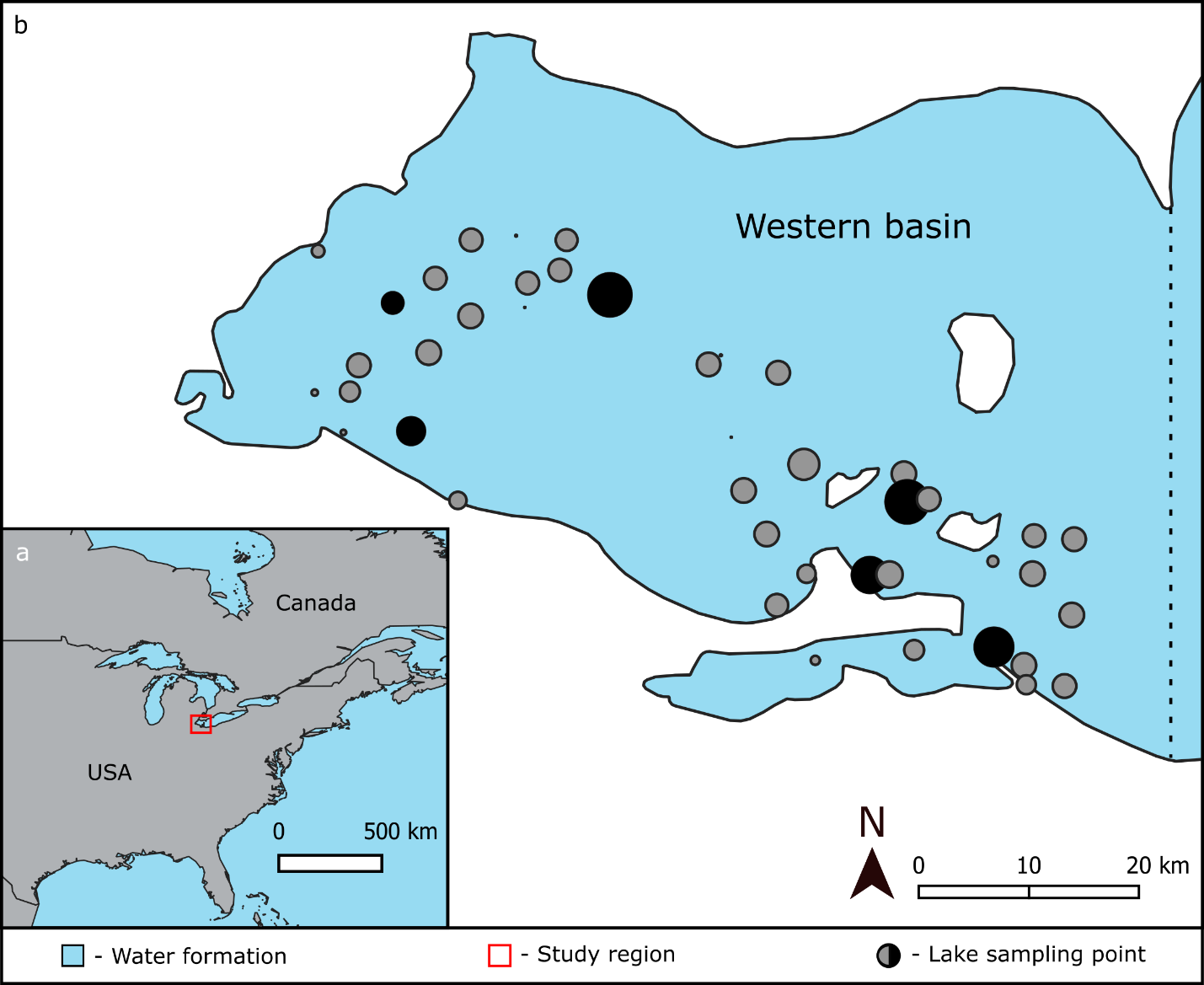


**Figure S1.1.** Primary trawl sampling locations (circles) in (a) the Great Lakes region of North America for (b) Lake Erie’s western basin. Trawl surveys were conducted by the ODNR-DOW in fall during 1969–2018. The border of Lake Erie’s western basin is denoted by a vertical dashed line. Black points indicate the six sites used for calculating the water transparency values. Points are sized relative to the number of years they have been sampled (50 years maximum).

**Section 2 – Temperature PCA**


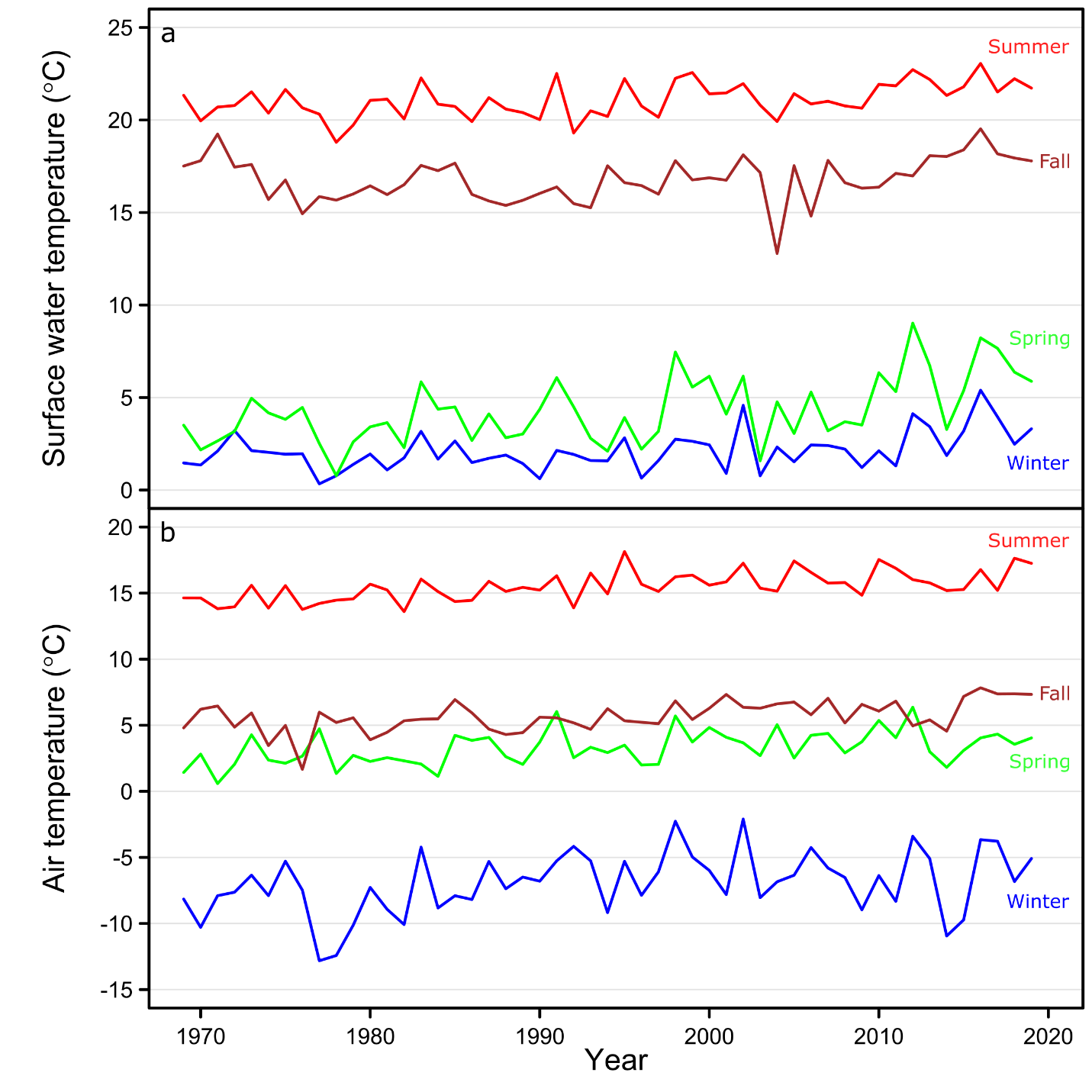


**Figure S2.1.** Temporal changes in mean seasonal (a) surface water and (b) air temperatures (°C) in Lake Erie (1969–2018). These temperatures were scaled by centering to their respective means and dividing by their standard deviations and then used as the response variables in the Principal Components Analysis shown in Fig. S2.2.


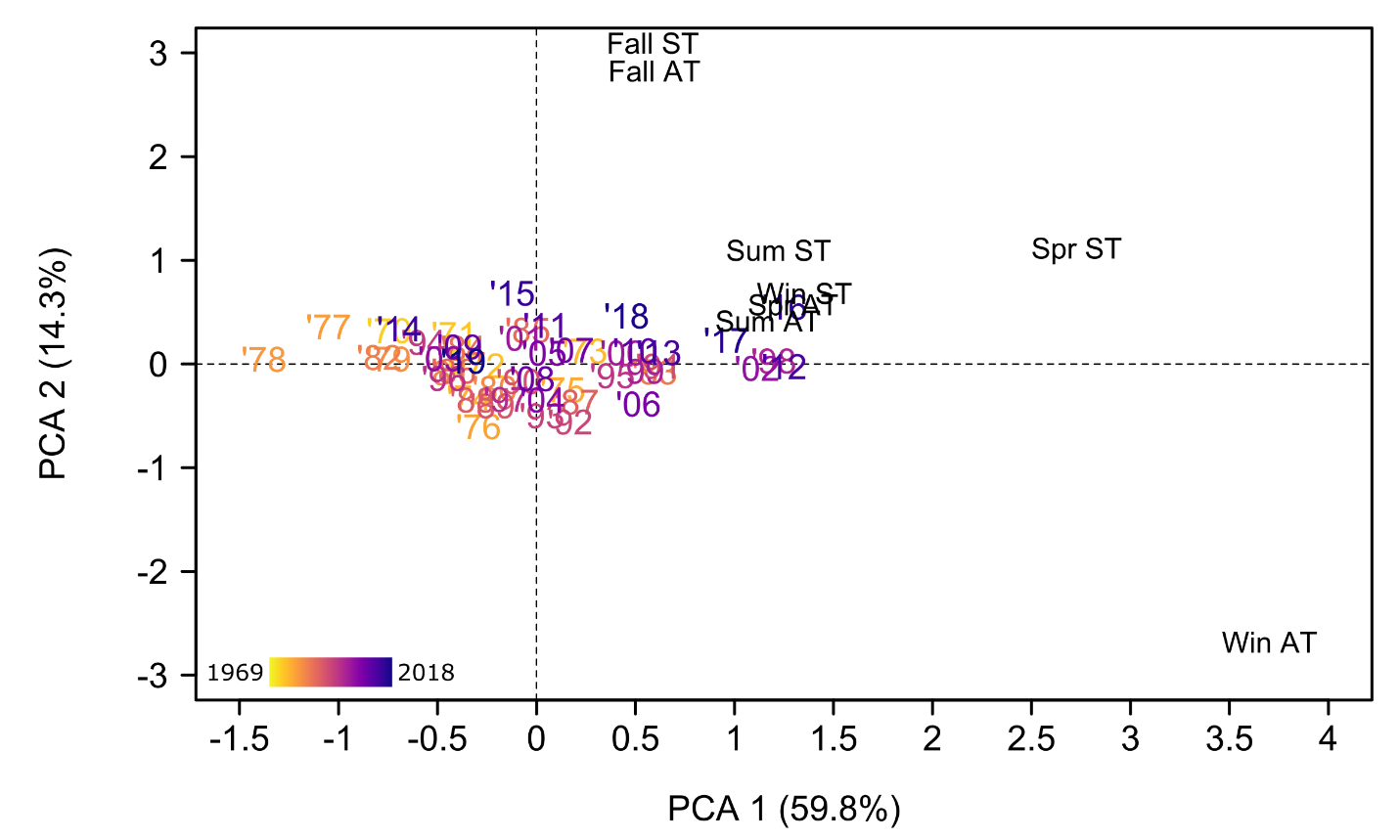


**Figure S2.2.** Principal Components Analysis (PCA) of mean annual Lake Erie surface water (‘ST’) and air (‘AT’) temperatures in the winter (‘Win’), spring (‘Spr’), summer (‘Sum’), and fall (‘Fall’) seasons (1969–2018). Points represent the seasonal temperatures in individual years and temporally progress across a color gradient from yellow to blue (orange and purple represent intermediate decades). Earlier years tend to be located on the left side of the ordination whereas later years tend to be located on the right side, indicating that seasonal surface water and air temperatures tended to increase during 1969–2018. Axis scores from PCA 1, which captures the majority of temperature variability (see Fig. S2.3), are therefore representative of these increases in seasonal temperatures through time (and also negatively correlate to the extent and duration of winter ice cover; Fig. S2.4).


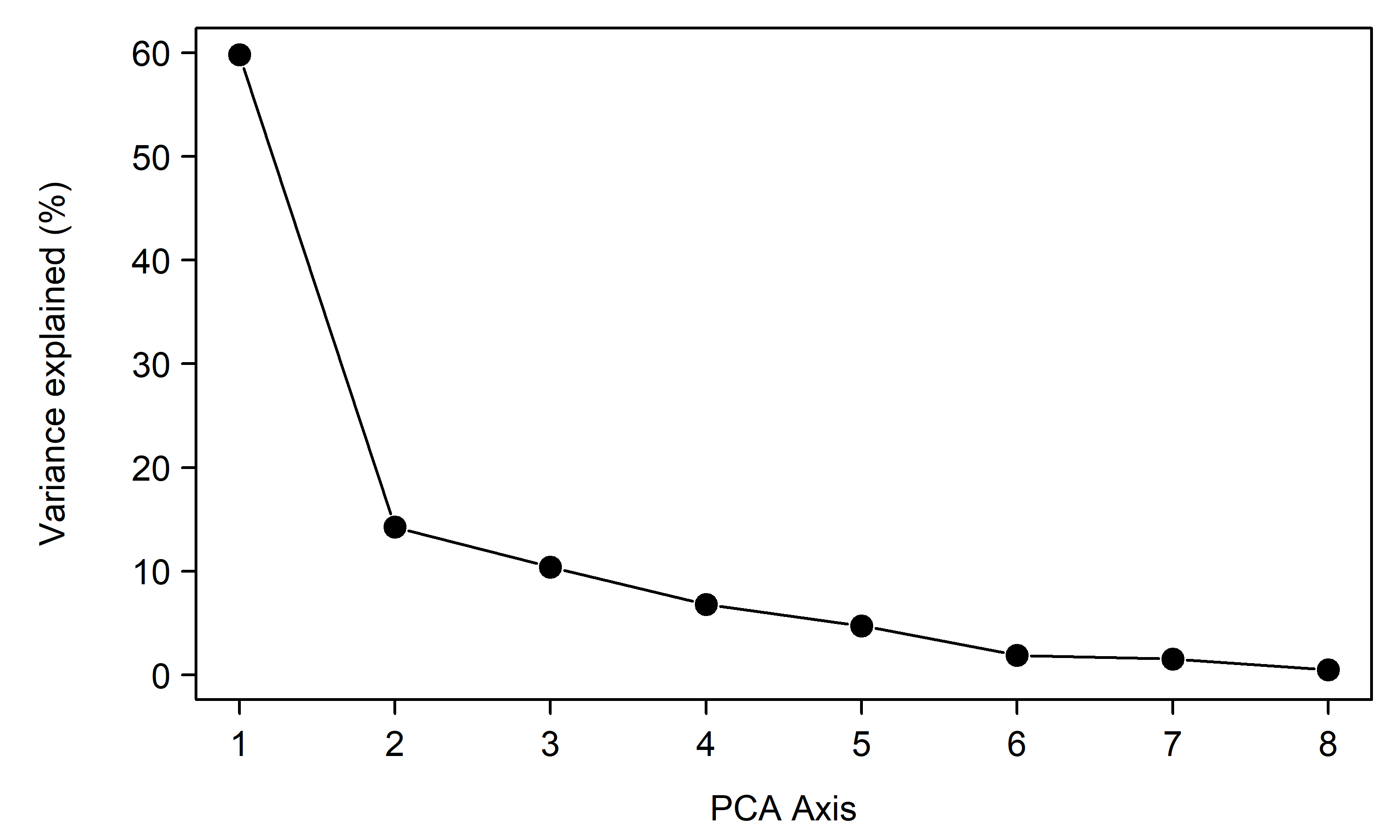


**Figure S2.3.** Total variance (%) explained by each axis in the Principal Components Analysis (PCA) of seasonal air and surface water temperatures. Most of the variability in seasonal temperatures is represented by PCA axis 1.


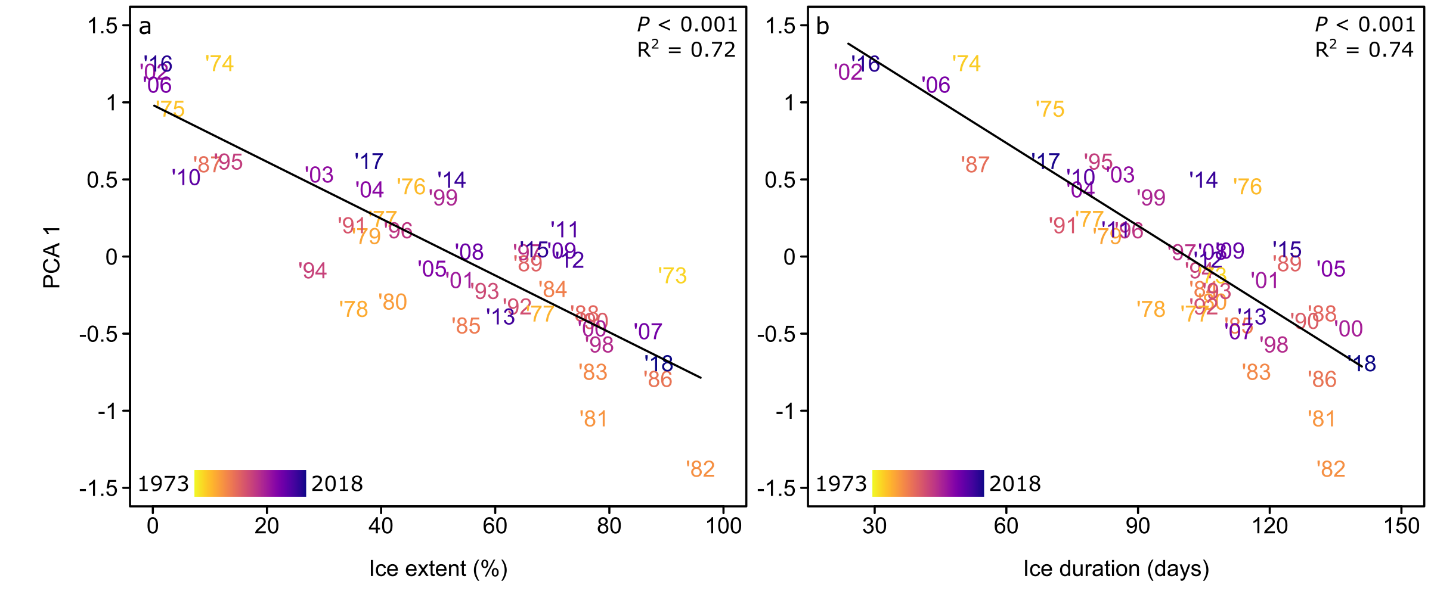


**Figure S2.4.** Relationship between temperature PCA axis 1 scores and the (a) maximum extent of yearly ice cover (% surface cover) and (b) duration of ice cover (days from Dec-May with >1% cover) in Lake Erie (1973–2018). Years with positive PCA 1 axis scores (i.e., warmer seasonal temperatures; Fig. S2.2) also tend to have lower ice extent and duration. Ice extent and duration data were obtained from the National Oceanic and Atmospheric Administration Great Lakes Environmental Research Laboratory (glerl.noaa.gov).

**Section 3 – GLS model results**

**Table S3.1.** Results of Generalized Least Squares Models (GLS). These models determined whether the relationship between younger walleye body size and older walleye abundance (‘Abundance’) changed in relation to changes in the lake environment (‘Environment’; two NMDS axes), total prey abundance, prey species composition (‘Prey species’; two NMDS axes), or prey trait composition (‘Prey traits’; two NMDS axes). We indicate which NMDS axes exhibited significant interactions in the ‘Interaction’ column and provide the associated statistics for this interaction based on log-likelihood ratio tests of models with versus without the interaction term.

| **Response** | **Predictors** | **Interaction** | ***L*** | ***P*** |
| --- | --- | --- | --- | --- |
| Age-1 body size | Abundance*Environment | NMDS 1 | 4.08 | 0.043 |
|  | Abundance*Prey abundance | – |  |  |
|  | Abundance*Prey species | NMDS 1 | 5.21 | 0.022 |
|  | Abundance*Prey traits | NMDS 1 | 7.10 | 0.008 |
| Age-2 body size | Abundance*Environment | – |  |  |
|  | Abundance*Prey abundance | – |  |  |
|  | Abundance*Prey species | NMDS 1 | 5.67 | 0.017 |
|  | Abundance*Prey traits | – |  |  |


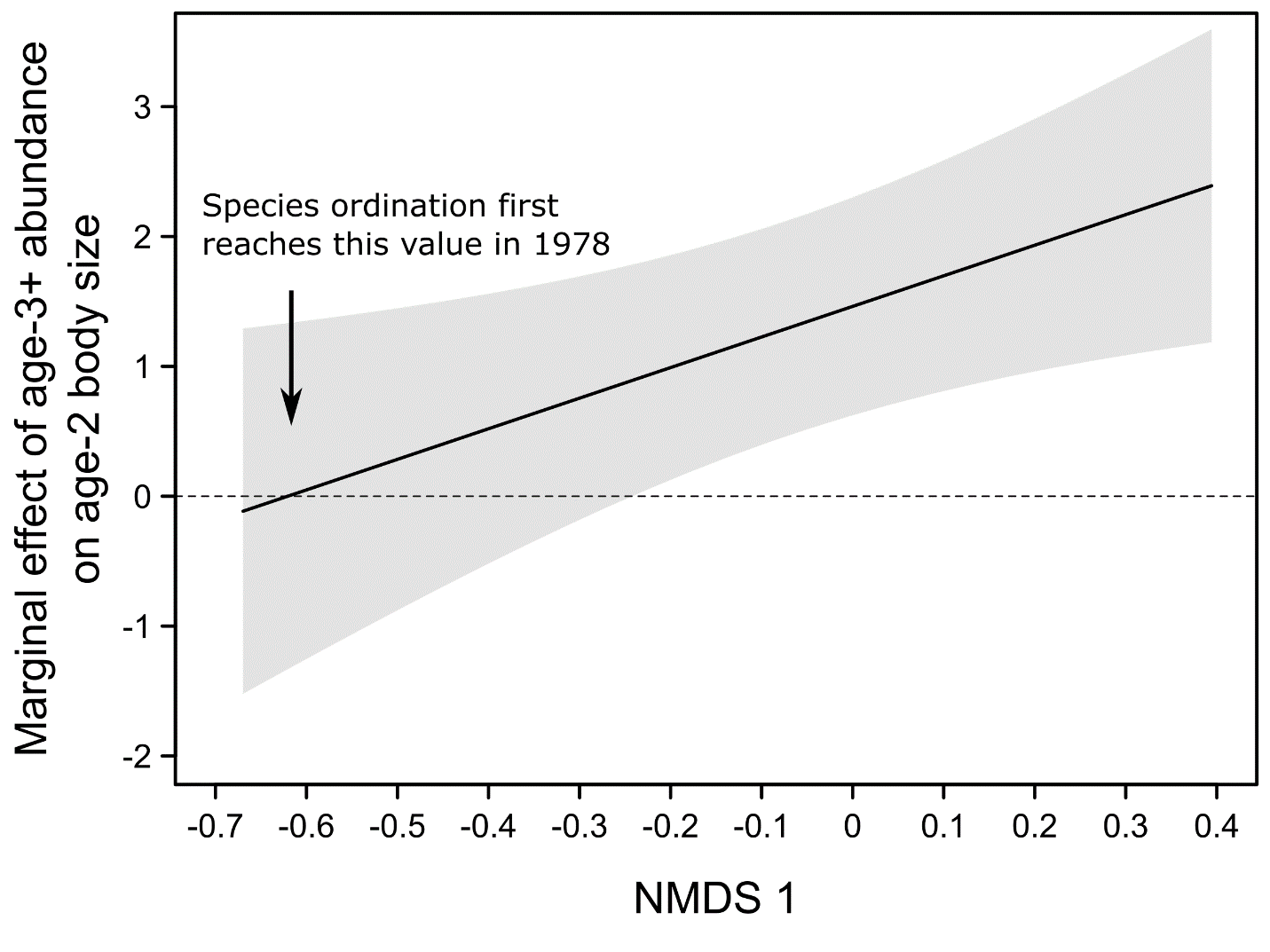


**Figure S3.1.** Marginal effect of older walleye (age-3+) abundance on younger (age-2) walleye body size conditioned on changes in prey-fish species composition. Negative marginal effects indicate a negative relationship, specifically that body size declines as abundance increases, whereas positive marginal effects indicate the opposite. The arrow is included to illustrate the approximate time period when the marginal effect switched from negative to positive based on comparisons to the ordination axis in Fig. 3d.

**Section 4 – List of functional traits**

**Table S4.** List of the functional traits for the prey-fish community of western Lake Erie (1969–2018). The data type of each trait (categorical or numeric), its respective trait values or range of values, and the source of these values are also provided. Trait values for each species are provided in a supplementary data appendix (Data S3).

| **Category** | **Trait** | **Data type** | **Values or range** | **Source(s)** |
| --- | --- | --- | --- | --- |
| Physiological tolerance | Sensitivity to environmental or anthropogenic stress | Categorical – ordinal | Intermediate, tolerant | 11 |
|  | Preferred or optimal temperature | Numeric | 11.5–30ºC | 11 |
| Functional niche | Primary adult diet composition | Categorical – nominal | Omnivore, planktivore, larval fish and invertebrates (‘Fish & Inverts’), piscivore | 12 |
|  | Primary habitat zone | Categorical – nominal | Benthic, pelagic | 13 |
|  | Spawn season | Categorical – ordinal | Spring (‘Spr’), spring and summer (‘SprSum’), summer (‘Sum’) | 11 |
| Predation | Maximum number of dorsal spines | Numeric | 0–14 | Wisconsin Fish ID program (seagrant.wisc.edu/fish-id) |
|  | Energy density | Numeric | 2,631–6,372 (J/g wet mass) | 5, 10 |

**Section 5 –** **Walleye length, body condition, maturity, and prey abundance**

The Ohio Department of Natural Resources-Division of Wildlife (ODNR-DOW) has been conducting walleye gill net surveys, from October to November, during 1974–2018 in Lake Erie’s western basin. These surveys include data on individual age, length-at-age (mm), weight (g; only available after 1978), and sexual condition. The annual mean length-at-age data we analyze in our main text is derived from the mean length of all younger (age-1 or age-2) individuals, which comprise the majority of the walleye captured in these surveys. We only used survey data collected up to 2015 owing to a gear change from kegged multifilament to kegged monofilament nets in 2016, which can affect the size of walleye captured. Walleye were aged using scales up to 2003 and then otoliths thereafter (see Madenjian et al. 2018 for a general size-at-age relationship for walleye in Lake Erie^10^).

Given the nature of this survey data and our results, we sought to answer several questions to confirm the validity of our methods and to further probe our conclusion regarding the shift in the density feedback through time. First, we confirmed that mean length-at-age values for younger walleye were representative of the central tendency of these populations (Fig. S5.1). Second, we quantified patterns in age-1 and age-2 lengths through time (Fig. S5.2) to test for the need to account for temporal autocorrelation and we examined the pre- versus post-breakpoint relationships to the number of growing degree days (Fig. S5.3). We found consistent temporal oscillations in the lengths of younger walleye and somewhat positive relationships to growing degree days so we controlled for these effects in our analyses. Third, a natural question that stemmed from our finding of a shift in the density feedback was whether we could find a signal of intraspecific competition in other metrics of younger walleye growth and life history, such as body condition (Fig. S5.4) or maturity (Fig. S5.5). We did not include these metrics in our main analyses because they tend to be mirrored by changes in mean length-at-age and these data were only available starting in the 1980s. We found positive or neutral relationships between younger walleye body condition/maturity and the size of the older (age-3+) walleye population after 1982. These results match our length-based findings and similarly indicate no density feedback between age-1/age-2 and age-3+ walleye during recent decades.


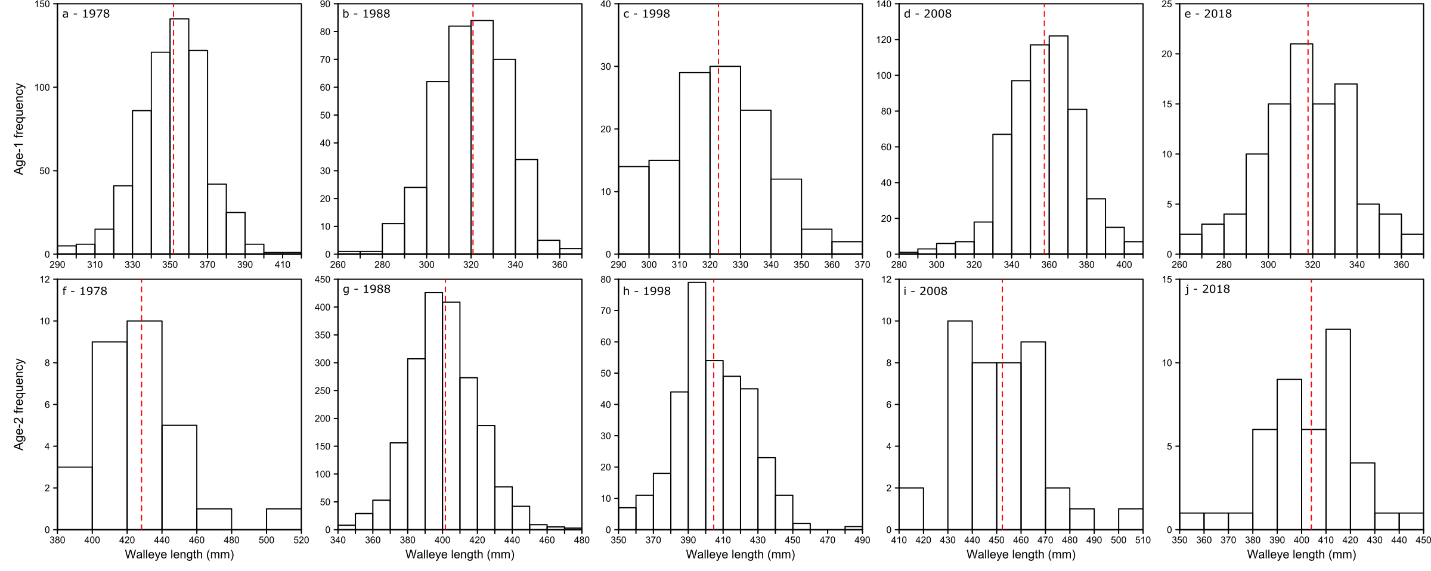


**Figure S5.1.** Individual length-at-age (mm) distributions for (a–e) age-1 and (f–j) age-2 walleye in Lake Erie during 1978 (age-1: *n* = 612; age-2: *n* = 29), 1988 (age-1: *n* = 503; age-2: *n* = 3,615), 1998 (age-1: *n* = 195; age-2: *n* = 720), 2008 (age-1: *n* = 1,231; age-2: *n* = 497), and 2018 (age-1: *n* = 146; age-2: *n* = 101). These distributions illustrate that annual mean length-at-age values (red dashed lines) are representative of the central tendency of the lengths of younger walleye in each year.


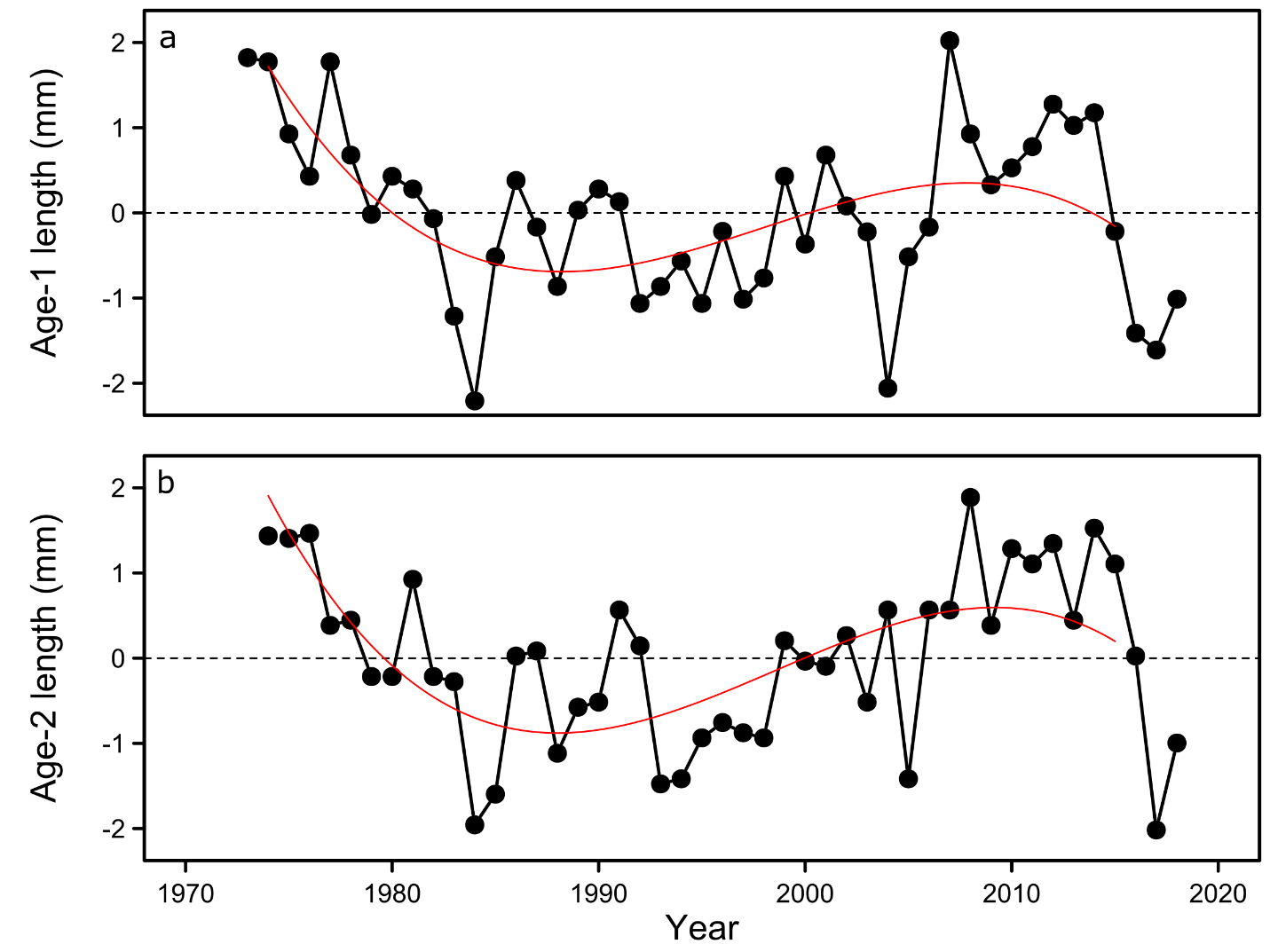


**Figure S5.2.** Change in mean (a) age-1 and (b) age-2 walleye lengths (mm) during 1974–2015. Lengths are centered to their means and 1 standard deviation for each age class to better visualize the temporal periodicity in the data. Best-fit lines (red) are drawn based on the predicted, linear relationship of length to the linear and quadratic terms for year. We included a first-order temporal autocorrelation structure in our models to control for these effects.


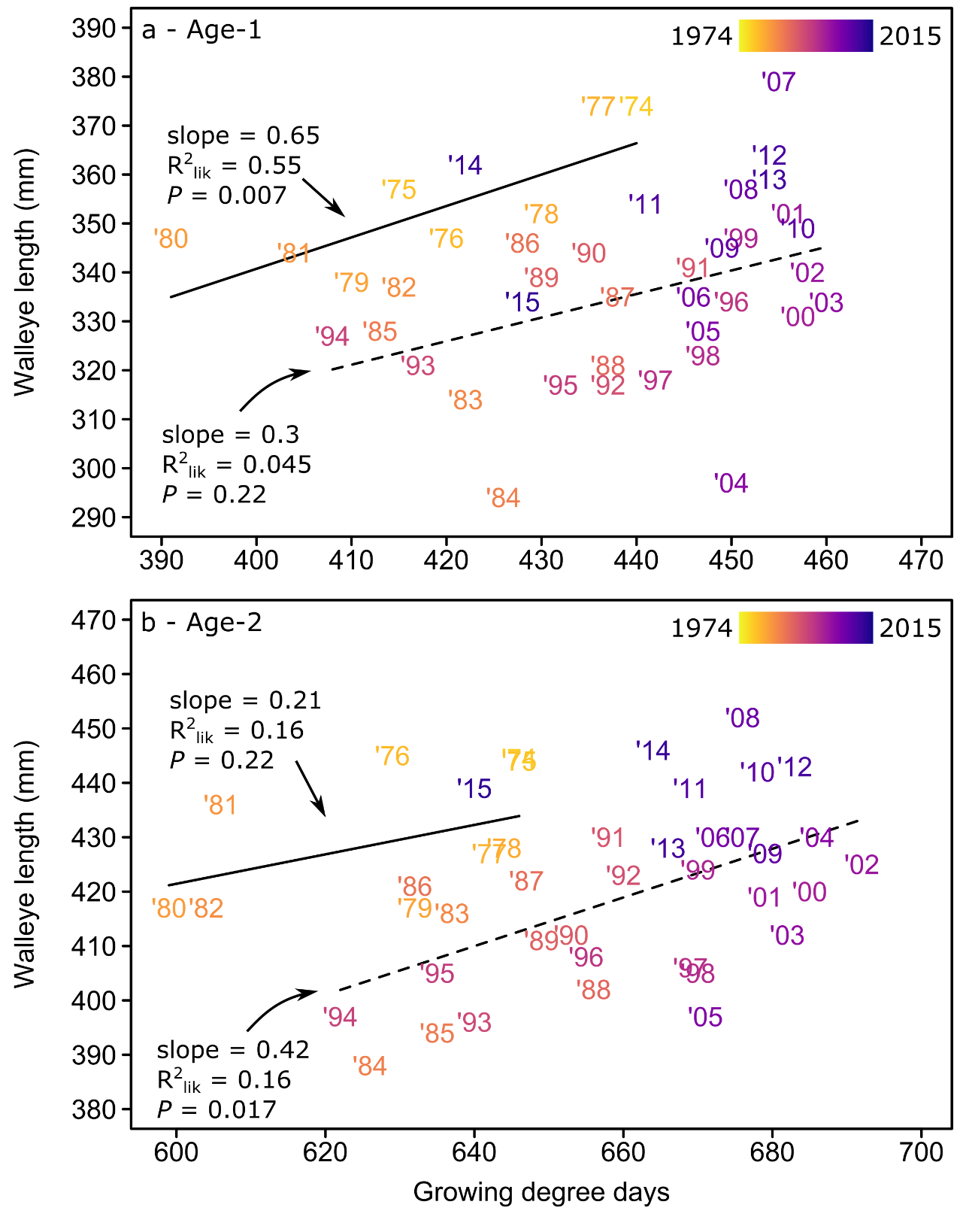


**Figure S5.3.** Relationship between (a) age-1 and (b) age-2 walleye lengths and the annual number of growing degree days (GDDs) for walleye in Lake Erie (1974–2015). GDDs were calculated following methods in Cheznik et al. (2014)^14^ based on the date of gill net surveys in each year, daily maximum and minimum air temperatures recorded at the Toledo, OH airport, and a T_0_ (temperature below which growth is effectively zero) of 5°C. Walleye lengths are somewhat positively related to the number of GDDs both before (solid lines) and after (dashed lines) the 1982 breakpoint discussed in the main text, indicating that annual differences in GDDs should be accounted for when modeling temporal changes in walleye lengths.

**
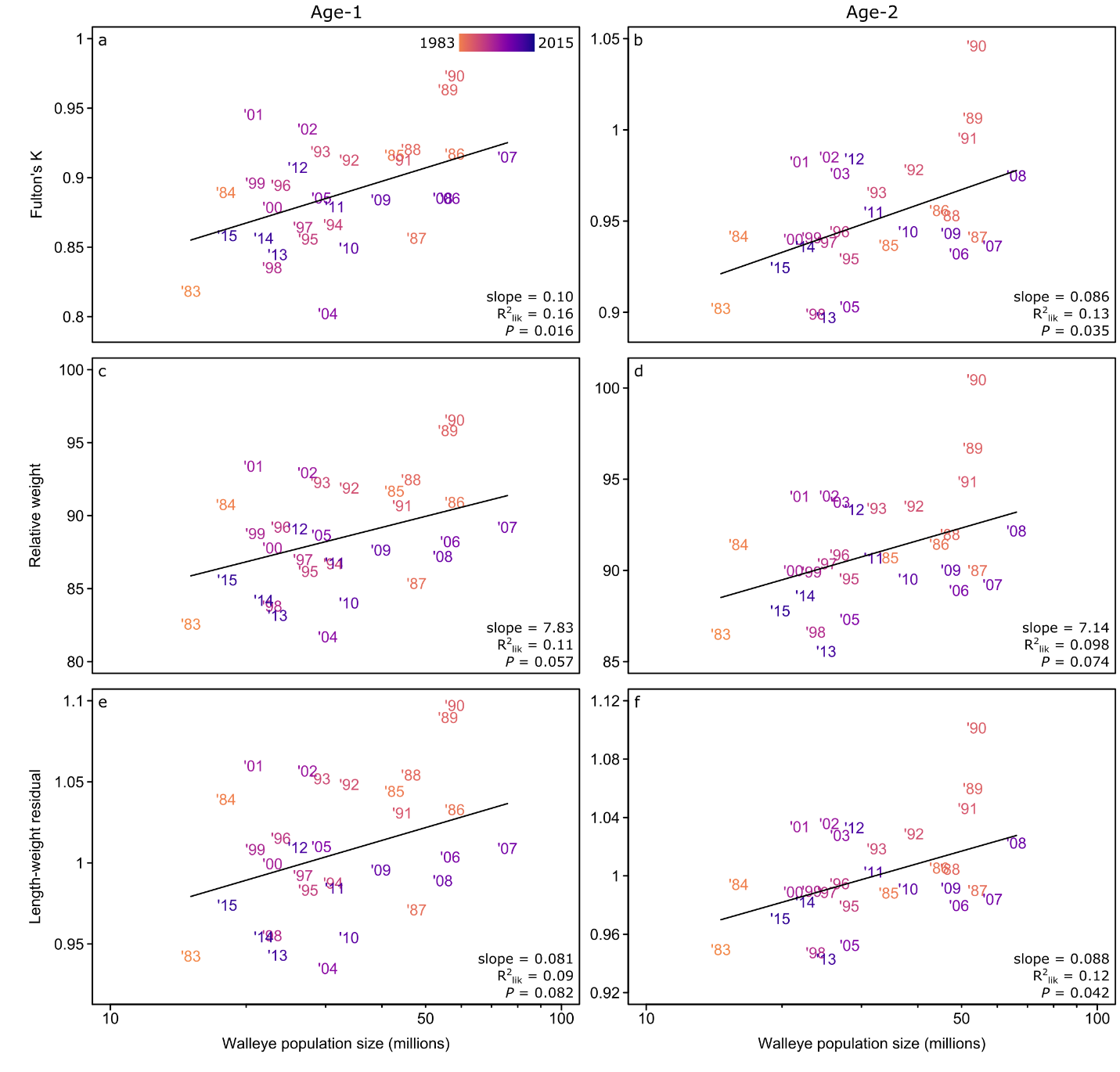
**

**Figure S5.4.** Relationship between (a, c, e) age-1 and (b, d, f) age-2 annual walleye body condition and the size of the older walleye population (millions of age-3+) in Lake Erie during 1983–2015. Body condition was calculated based on annual mean walleye length-at-age (mm) and weight (g) from ODNR-DOW fall gill net surveys using (a–b) Fulton’s K^15^, (c–d) relative weight^16^, and the residuals of a length-weight regression. All measurements of condition show a neutral or positive relationship with the size of the older walleye population, indicating no density feedback between older and younger individuals.

**
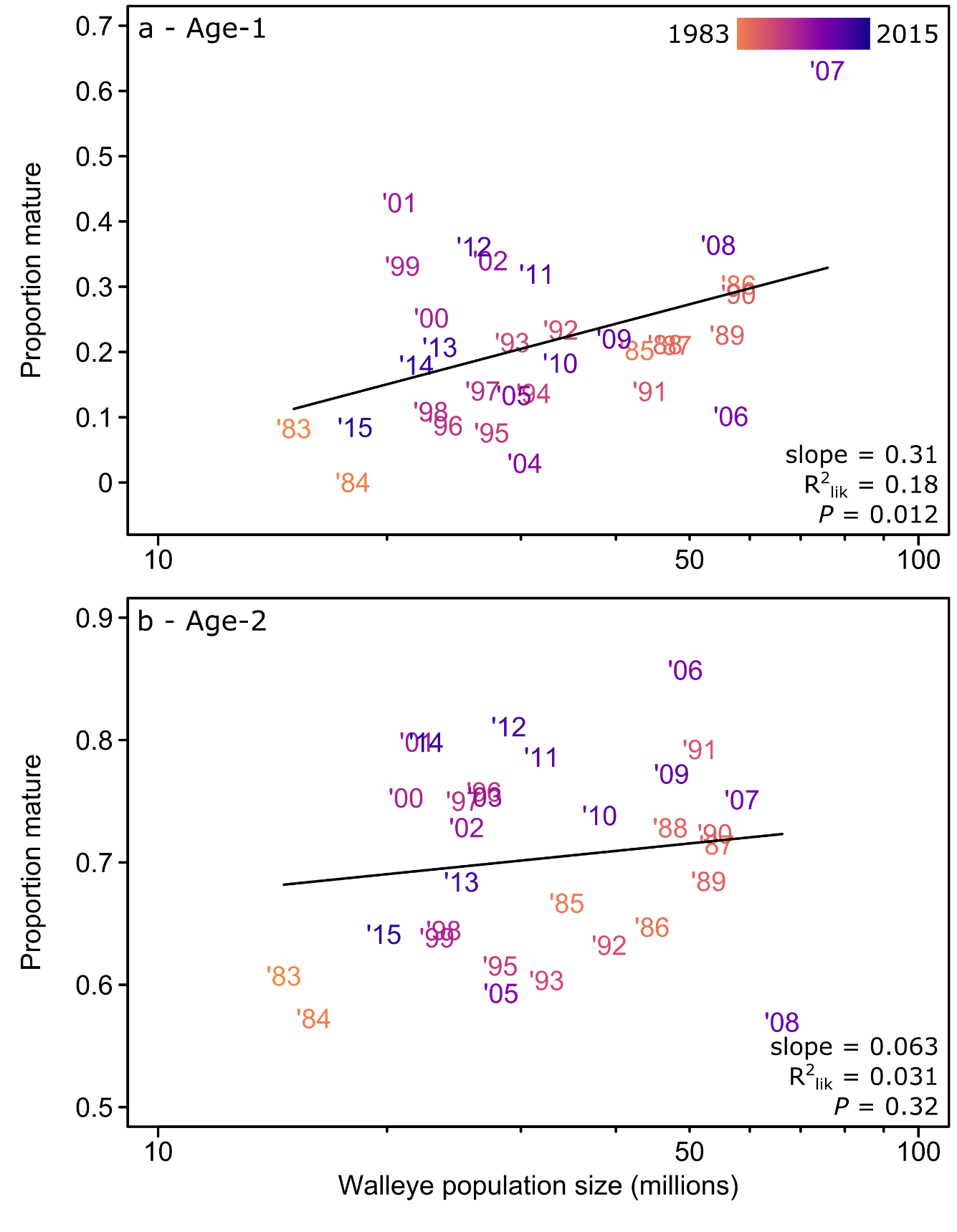
**

**Figure S5.5.** Relationship between the annual proportion of the (a) age-1 and (b) age-2 walleye population that is sexually mature and the size of the older walleye population (millions of age-3+) in Lake Erie (1983–2015). Maturity tends to be unrelated or positively related to the size of the older walleye population, indicating no density feedback between older and younger individuals.

**Section 6 –** **Relationship between younger walleye length and total prey-fish abundance**


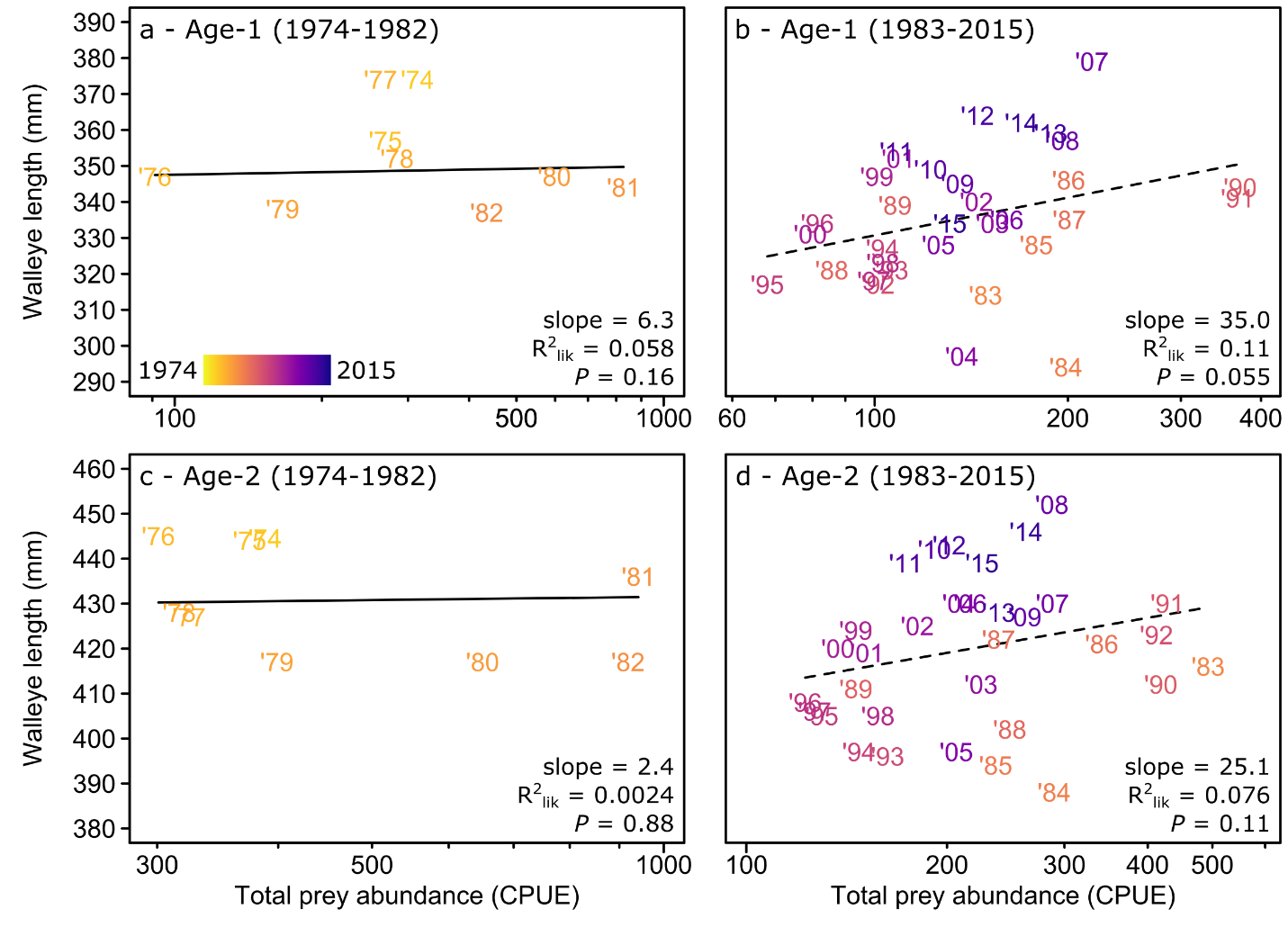


**Figure S6.1.** Relationship between the mean (a–b) age-1 and (c–d) age-2 walleye lengths (mm) and total prey-fish abundance (catch-per-unit-effort; CPUE) both (a, c) before and (b, d) after the 1982 breakpoint. The length-at-age of younger walleye became somewhat more positively related to prey-fish abundance after 1982.

**References**

1. Vandergoot, C. S., Cook, H. A., Thomas, M. V., Einhouse, D. E. & Murray, C. Status of walleye in western Lake Erie, 1985-2006. in *Status of walleye in the Great Lakes: proceedings of the 2006 Symposium* vol. Technical report 69 123–150 (Great Lakes Fishery Commission, Ann Arbor, Michigan, USA, 2010).

2. Wang, H.-Y. *et al.* Movement of walleyes in Lakes Erie and St. Clair inferred from tag return and fisheries data. *Trans. Am. Fish. Soc.* **136**, 539–551 (2007).

3. Uphoff, C. S., Schoenebeck, C. W., Koupal, K. D., Pope, K. L. & Wyatt Hoback, W. Age-0 walleye Sander vitreus display length-dependent diet shift to piscivory. *J. Freshw. Ecol.* **34**, 27–36 (2019).

4. Knight, R. L., Margraf, F. J. & Carline, R. F. Piscivory by walleyes and yellow perch in western Lake Erie. *Trans. Am. Fish. Soc.* **113**, 677–693 (1984).

5. Kershner, M. W. Walleye predatory demand and prey supply in Lake Erie: exploring environmental variation and its historical effects. (The Ohio State University, 1998).

6. Tyson, J. T., Johnson, T. B., Knight, C. T. & Bur, M. T. Intercalibration of research survey vessels on Lake Erie. *North Am. J. Fish. Manag.* **26**, 559–570 (2006).

7. Hartman, K. J. & Margraf, F. J. Effects of prey and predator abundances on prey consumption and growth of walleyes in western Lake Erie. *Trans. Am. Fish. Soc.* **121**, 245–260 (1992).

8. Knight, R. L. & Vondracek, B. Changes in prey fish populations in western Lake Erie, 1969–88, as related to walleye, *Stizostedion vitreum*, predation. *Can. J. Fish. Aquat. Sci.* **50**, 1289–1298 (1993).

9. Johnson, T. B., Bunnell, D. B. & Knight, C. T. A potential new energy pathway in central Lake Erie: the round goby connection. *J. Gt. Lakes Res.* **31**, 238–251 (2005).

10. Madenjian, C. P. *et al.* Temperature regimes, growth, and food consumption for female and male adult walleye in Lake Huron and Lake Erie: a bioenergetics analysis. *Can. J. Fish. Aquat. Sci.* **75**, 1573–1586 (2018).

11. Eakins, R. J. Ontario freshwater fishes life history database. (2020).

12. Froese, R. & Pauly, D. FishBase. (2019).

13. Frimpong, E. A. & Angermeier, P. L. Fish Traits: a database of ecological and life-history traits of freshwater fishes of the United States. *Fisheries* **34**, 487–495 (2009).

14. Chezik, K. A., Lester, N. P. & Venturelli, P. A. Fish growth and degree-days I: selecting a base temperature for a within-population study. *Can. J. Fish. Aquat. Sci.* **71**, 47–55 (2014).

15. Ricker, W. E. Computation and interpretation of biological statistics of fish populations. *Bull Fish Res Bd Can* **191**, 1–382 (1975).

16. Wege, G. J. & Anderson, R. O. Relative weight (Wr): a new index of condition for largemouth bass. *New Approaches Manag. Small Impound. Am. Fish. Soc. North Cent. Div. Spec. Publ.* **5**, 79–91 (1978).
